# Tea polyphenol mediated *CsMYB77* regulation of *CsPOD44* to promote tea plant (*Camellia sinensis*) root drought resistance

**DOI:** 10.1093/hr/uhaf048

**Published:** 2025-02-18

**Authors:** Rong Xu, Chenyu Shao, Yuqi Luo, Biao Zhou, Qian Zhu, Shuqi Qiu, Zhonghua Liu, Shuoqian Liu, Chengwen Shen

**Affiliations:** Key Laboratory of Tea Science of Ministry of Education, Hunan Agricultural University, No. 1 Nongda Road, Furong District, Changsha, Hunan 410128, China; National Research Center of Engineering and Technology for Utilization of Botanical Functional Ingredients, Hunan Agricultural University, No. 1 Nongda Road, Furong District, Changsha, Hunan 410128, China; Co-Innovation Center of Education Ministry for Utilization of Botanical Functional Ingredients, Hunan Agricultural University, No. 1 Nongda Road, Furong District, Changsha, Hunan 410128, China; Key Laboratory for Evaluation and Utilization of Gene Resources of Horticultural Crops, Ministry of Agriculture and Rural Affairs of China, Hunan Agricultural University, No. 1 Nongda Road, Furong District, Changsha, Hunan 410128, China; Yuelushan Laboratory, No. 1 Nongda Road, Furong District, Changsha, Hunan 410128, China; National Key Laboratory for Tea Plant Germplasm Innovation and Resource Utilization, No. 1 Nongda Road, Furong District, Changsha, Hunan 410128, China; Key Laboratory of Tea Science of Ministry of Education, Hunan Agricultural University, No. 1 Nongda Road, Furong District, Changsha, Hunan 410128, China; National Research Center of Engineering and Technology for Utilization of Botanical Functional Ingredients, Hunan Agricultural University, No. 1 Nongda Road, Furong District, Changsha, Hunan 410128, China; Co-Innovation Center of Education Ministry for Utilization of Botanical Functional Ingredients, Hunan Agricultural University, No. 1 Nongda Road, Furong District, Changsha, Hunan 410128, China; Key Laboratory for Evaluation and Utilization of Gene Resources of Horticultural Crops, Ministry of Agriculture and Rural Affairs of China, Hunan Agricultural University, No. 1 Nongda Road, Furong District, Changsha, Hunan 410128, China; Yuelushan Laboratory, No. 1 Nongda Road, Furong District, Changsha, Hunan 410128, China; National Key Laboratory for Tea Plant Germplasm Innovation and Resource Utilization, No. 1 Nongda Road, Furong District, Changsha, Hunan 410128, China; Key Laboratory of Tea Science of Ministry of Education, Hunan Agricultural University, No. 1 Nongda Road, Furong District, Changsha, Hunan 410128, China; National Research Center of Engineering and Technology for Utilization of Botanical Functional Ingredients, Hunan Agricultural University, No. 1 Nongda Road, Furong District, Changsha, Hunan 410128, China; Co-Innovation Center of Education Ministry for Utilization of Botanical Functional Ingredients, Hunan Agricultural University, No. 1 Nongda Road, Furong District, Changsha, Hunan 410128, China; Key Laboratory for Evaluation and Utilization of Gene Resources of Horticultural Crops, Ministry of Agriculture and Rural Affairs of China, Hunan Agricultural University, No. 1 Nongda Road, Furong District, Changsha, Hunan 410128, China; Yuelushan Laboratory, No. 1 Nongda Road, Furong District, Changsha, Hunan 410128, China; National Key Laboratory for Tea Plant Germplasm Innovation and Resource Utilization, No. 1 Nongda Road, Furong District, Changsha, Hunan 410128, China; Key Laboratory of Tea Science of Ministry of Education, Hunan Agricultural University, No. 1 Nongda Road, Furong District, Changsha, Hunan 410128, China; National Research Center of Engineering and Technology for Utilization of Botanical Functional Ingredients, Hunan Agricultural University, No. 1 Nongda Road, Furong District, Changsha, Hunan 410128, China; Co-Innovation Center of Education Ministry for Utilization of Botanical Functional Ingredients, Hunan Agricultural University, No. 1 Nongda Road, Furong District, Changsha, Hunan 410128, China; Key Laboratory for Evaluation and Utilization of Gene Resources of Horticultural Crops, Ministry of Agriculture and Rural Affairs of China, Hunan Agricultural University, No. 1 Nongda Road, Furong District, Changsha, Hunan 410128, China; Yuelushan Laboratory, No. 1 Nongda Road, Furong District, Changsha, Hunan 410128, China; National Key Laboratory for Tea Plant Germplasm Innovation and Resource Utilization, No. 1 Nongda Road, Furong District, Changsha, Hunan 410128, China; Key Laboratory of Tea Science of Ministry of Education, Hunan Agricultural University, No. 1 Nongda Road, Furong District, Changsha, Hunan 410128, China; National Research Center of Engineering and Technology for Utilization of Botanical Functional Ingredients, Hunan Agricultural University, No. 1 Nongda Road, Furong District, Changsha, Hunan 410128, China; Co-Innovation Center of Education Ministry for Utilization of Botanical Functional Ingredients, Hunan Agricultural University, No. 1 Nongda Road, Furong District, Changsha, Hunan 410128, China; Key Laboratory for Evaluation and Utilization of Gene Resources of Horticultural Crops, Ministry of Agriculture and Rural Affairs of China, Hunan Agricultural University, No. 1 Nongda Road, Furong District, Changsha, Hunan 410128, China; Yuelushan Laboratory, No. 1 Nongda Road, Furong District, Changsha, Hunan 410128, China; National Key Laboratory for Tea Plant Germplasm Innovation and Resource Utilization, No. 1 Nongda Road, Furong District, Changsha, Hunan 410128, China; Key Laboratory of Tea Science of Ministry of Education, Hunan Agricultural University, No. 1 Nongda Road, Furong District, Changsha, Hunan 410128, China; National Research Center of Engineering and Technology for Utilization of Botanical Functional Ingredients, Hunan Agricultural University, No. 1 Nongda Road, Furong District, Changsha, Hunan 410128, China; Co-Innovation Center of Education Ministry for Utilization of Botanical Functional Ingredients, Hunan Agricultural University, No. 1 Nongda Road, Furong District, Changsha, Hunan 410128, China; Key Laboratory for Evaluation and Utilization of Gene Resources of Horticultural Crops, Ministry of Agriculture and Rural Affairs of China, Hunan Agricultural University, No. 1 Nongda Road, Furong District, Changsha, Hunan 410128, China; Yuelushan Laboratory, No. 1 Nongda Road, Furong District, Changsha, Hunan 410128, China; National Key Laboratory for Tea Plant Germplasm Innovation and Resource Utilization, No. 1 Nongda Road, Furong District, Changsha, Hunan 410128, China; Key Laboratory of Tea Science of Ministry of Education, Hunan Agricultural University, No. 1 Nongda Road, Furong District, Changsha, Hunan 410128, China; National Research Center of Engineering and Technology for Utilization of Botanical Functional Ingredients, Hunan Agricultural University, No. 1 Nongda Road, Furong District, Changsha, Hunan 410128, China; Co-Innovation Center of Education Ministry for Utilization of Botanical Functional Ingredients, Hunan Agricultural University, No. 1 Nongda Road, Furong District, Changsha, Hunan 410128, China; Key Laboratory for Evaluation and Utilization of Gene Resources of Horticultural Crops, Ministry of Agriculture and Rural Affairs of China, Hunan Agricultural University, No. 1 Nongda Road, Furong District, Changsha, Hunan 410128, China; Yuelushan Laboratory, No. 1 Nongda Road, Furong District, Changsha, Hunan 410128, China; National Key Laboratory for Tea Plant Germplasm Innovation and Resource Utilization, No. 1 Nongda Road, Furong District, Changsha, Hunan 410128, China; Key Laboratory of Tea Science of Ministry of Education, Hunan Agricultural University, No. 1 Nongda Road, Furong District, Changsha, Hunan 410128, China; National Research Center of Engineering and Technology for Utilization of Botanical Functional Ingredients, Hunan Agricultural University, No. 1 Nongda Road, Furong District, Changsha, Hunan 410128, China; Co-Innovation Center of Education Ministry for Utilization of Botanical Functional Ingredients, Hunan Agricultural University, No. 1 Nongda Road, Furong District, Changsha, Hunan 410128, China; Key Laboratory for Evaluation and Utilization of Gene Resources of Horticultural Crops, Ministry of Agriculture and Rural Affairs of China, Hunan Agricultural University, No. 1 Nongda Road, Furong District, Changsha, Hunan 410128, China; Yuelushan Laboratory, No. 1 Nongda Road, Furong District, Changsha, Hunan 410128, China; National Key Laboratory for Tea Plant Germplasm Innovation and Resource Utilization, No. 1 Nongda Road, Furong District, Changsha, Hunan 410128, China; Key Laboratory of Tea Science of Ministry of Education, Hunan Agricultural University, No. 1 Nongda Road, Furong District, Changsha, Hunan 410128, China; National Research Center of Engineering and Technology for Utilization of Botanical Functional Ingredients, Hunan Agricultural University, No. 1 Nongda Road, Furong District, Changsha, Hunan 410128, China; Co-Innovation Center of Education Ministry for Utilization of Botanical Functional Ingredients, Hunan Agricultural University, No. 1 Nongda Road, Furong District, Changsha, Hunan 410128, China; Key Laboratory for Evaluation and Utilization of Gene Resources of Horticultural Crops, Ministry of Agriculture and Rural Affairs of China, Hunan Agricultural University, No. 1 Nongda Road, Furong District, Changsha, Hunan 410128, China; Yuelushan Laboratory, No. 1 Nongda Road, Furong District, Changsha, Hunan 410128, China; National Key Laboratory for Tea Plant Germplasm Innovation and Resource Utilization, No. 1 Nongda Road, Furong District, Changsha, Hunan 410128, China

## Abstract

Drought stress significantly alters the metabolic homeostasis of tea plants; however, few studies have examined the role of specific metabolites, particularly tea polyphenols, in drought resistance. This study reveals that the tea polyphenol content in drought-tolerant tea cultivars tends to increase under drought conditions. Notably, in environments characterized by staged and repeated drought, changes in tea polyphenol are significantly positively correlated with drought resistance. To investigate this further, we irrigated the roots with exogenous tea polyphenols before subjecting the plants to drought. Our findings indicated that the absorptive roots of the experimental group exhibited enhanced development, improved cellular integrity, and a significant increase in peroxidase activity. A comprehensive analysis of the transcriptome and metabolome revealed that tea polyphenols are closely associated with the phenylpropanoid metabolism pathway. Notably, *CsMYB77* and *CsPOD44* genes were identified as highly correlated with this pathway. Overexpression experiments in *Arabidopsis thaliana* demonstrated that *CsMYB77* promotes the expression of phenylpropanoid pathway genes, thereby enhancing drought resistance. Conversely, antisense oligonucleotide silencing of *CsMYB77* decreased drought resistance in tea plants. Additional experiments, including yeast one-hybrid assays, luciferase complementation imaging, dual-luciferase assays, and electrophoretic mobility shift assays, confirmed that *CsMYB77* positively regulates the expression of *CsPOD44*. In summary, our findings indicate that the differences in drought tolerance among tea cultivars are closely linked to phenylpropanoid metabolism. Specifically, tea polyphenols may mediate the regulatory network involving *CsMYB77* and *CsPOD44*, thereby enhancing stress resistance by promoting root development. This study offers new insights into the breeding of drought-resistant tea cultivars.

## Introduction

Over the past decade, global crop yield losses attributable to drought have been estimated at approximately US$30 billion [1]. The frequency and intensity of drought conditions significantly impede the growth and development of various crops [2]. Tea plants (*Camellia sinensis*), which thrive in humid environments, are particularly vulnerable to drought stress, which can substantially reduce both yield and quality of tea leaves [3]. Studies indicate that global reductions in tea yield due to drought can range from 14% to 33%, while seedling mortality rates may reach between 6% and 19% [4]. Given the tea plant’s significant economic importance globally [5], there is an urgent need for strategies to mitigate drought-induced damage.

Different scenarios of drought stress induce physiological, metabolic, and molecular changes in plants, impacting their growth, development, and survival. Repeated drought events inhibit root growth in horticultural plants, particularly at the root tips and root hair zones, which diminishes the plant’s capacity to absorb water and nutrients [6]. Under intermittent drought conditions, the expression of transcripts associated with photosynthesis and metabolic processes is reduced, further influencing the growth and functionality of the root system, including root morphology and absorptive capacity [7]. Additionally, periodic drought alters the expression of genes related to plant antioxidant defense and osmotic regulation, thereby impeding overall plant growth and development [8]. Research has demonstrated that a robust root system enhances drought resistance in tea plants. However, drought stress can induce elongation of the primary root and accelerate xylem production to compensate for water deficits [9]. While the tea plant root system plays a critical role in resilience to drought stress, the mechanisms governing its stress resistance remain insufficiently understood.

The synthesis of tea polyphenols is sensitive to environmental disturbances, including high temperatures, cold, drought, salt-alkali conditions, and other abiotic stresses. The accumulation of tea polyphenols is closely linked to the stress resistance of plants [10]. Notably, tea plants exhibit an exceptionally high concentration of polyphenols compared to other species, with polyphenols constituting approximately 18% to 36% of the dry weight of tea leaves [11]. These compounds are primarily synthesized through the phenylpropanoid and flavonoid pathways [12]. The principal polyphenolic compound, catechin, is a fundamental structural component of α-phenyl-benzopyran and is an essential quality indicator in tea [13].

Recent studies have indicated that the accumulation of catechins is closely linked to plant stress resistance [14]. Many biosynthetic genes associated with catechins are induced under stressful conditions, leading to increased catechin levels when plants are exposed to abiotic stress. A typical response among plants facing abiotic stress is producing and accumulating reactive oxygen species (ROS) [15]. Tea polyphenols play a crucial role as antioxidant substances in mitigating stress; they stimulate the activity of antioxidant enzymes, eliminate excess ROS, and enhance plant stress resistance [16]. Furthermore, some studies have shown that exogenous catechins can bolster the free radical scavenging system, effectively reducing the concentrations of H_2_O_2_ and O^2−^ and significantly alleviating stress damage to plant leaves and roots [17]. Exogenous EGCG has also decreased ROS accumulation, thereby protecting the photosynthetic machinery. The reduction of ROS accumulation induced by EGCG is primarily attributed to the enhancement of the activities of peroxidase (POD), superoxide dismutase (SOD), catalase (CAT), and ascorbate peroxidase (APX) [16]. While studies have demonstrated that tea polyphenols possess potential drought resistance, the mechanisms by which these compounds enhance the drought resistance of tea plant roots through the regulation of internal metabolic pathways remain unclear. This study aims to address this knowledge gap by exploring the regulatory mechanisms of tea polyphenols on the drought resistance of tea plant roots. This will be achieved through a comprehensive analysis of phenotypes, enzyme activities, physiological and biochemical indicators, and transcriptome and metabolome data.

The MYB transcription factor family, one of the largest and most prominent gene families in plants, plays a critical role in regulating root development and responses to abiotic stress. MYB proteins contain a highly conserved N-terminal DNA-binding domain (DBD) [18]. For instance, research on *IbMYB73* has shown that it inhibits ABA-dependent adventitious root growth, increasing the sensitivity of sweet potato to drought stress by targeting *IbGER5*  *[19]*. In *Arabidopsis thaliana*, *MYB30* loss-of-function mutants have exhibited significant increases in root hair length [20], while mutations in *OsMYB60* impair epidermal wax biosynthesis, reducing drought tolerance in rice [21]. Similarly, *GhMYB102* has been shown to regulate the expression of *GhNCED1* and *GhZAT10*, enhancing ABA synthesis and improving drought tolerance in cotton [22]. Furthermore, MYB factors have been shown to regulate key antioxidant enzymes, including peroxidase (POD), superoxide dismutase (SOD), and ascorbate peroxidase (APX), which are crucial for ROS scavenging under stress conditions [23].

In this study, we investigated the effects of tea polyphenols on tea plant phenotypes, enzyme activities, and physiological and biochemical indicators, finding that tea polyphenols effectively mitigate drought-induced damage to tea plant roots. Transcriptome-metabolome data analysis identified two genes, *CsMYB77* and *CsPOD44*, within the phenylpropanoid pathway. Overexpression experiments in Arabidopsis and antisense oligonucleotide studies demonstrated that *CsMYB77* contributes to drought resistance. A series of molecular studies also indicated that *CsMYB77* regulates *CsPOD44*, enhancing its transcriptional expression. Our findings suggest that tea polyphenols modulate *CsPOD44* by mediating *CsMYB77* in the phenylpropanoid pathway, thereby improving the drought resistance of tea plant roots.

## Results

### Differences in tea polyphenol metabolic flux among tea cultivars mediate different drought resistance

Previous research has demonstrated a significant positive correlation between changes in the Fv/Fm ratio of tea plant leaves, and the concentration of tea polyphenols in response to staged, repeated drought conditions (Fig. S3). The Fv/Fm ratio is commonly utilized as an indicator of plant stress. Thus, it is hypothesized that tea polyphenols may enhance the drought tolerance of tea plants [24]. This study found that, in the control group, the leaves of five tea plant cultivars exhibited robust growth and a shiny appearance. However, after 9 days of drought stress treatment, notable differences emerged between the buds and leaves of the five cultivars. The leaves of the non-drought-resistant cultivars ‘Baojing Huangjincha1’ and ‘Bixiangzao’ exhibited varying degrees of wilting, curling, drooping, and browning. In contrast, the leaves of the drought-resistant cultivars ‘9808’, ‘Zhuyeqi’, and ‘Taoyuandaye’ displayed no significant phenotypic differences compared to the control group. This indicates that ‘Baojing Huangjincha1’ and ‘Bixiangzao’ are more susceptible to substantial damage under drought stress, while ‘9808’, ‘Taoyuandaye’, and ‘Zhuyeqi’ demonstrate more robust drought tolerance (Fig. S4A). Changes in relative conductivity can intuitively reflect the degree of stress experienced by plants. Consequently, we also measured variations in relative conductivity; the results indicated that after 9 days of drought treatment, the relative conductivity of the leaves of ‘Bixiangzao’ was significantly higher than that of the other four cultivars, reaching a maximum value of 51.98%. This was followed by ‘Baojing Huangjincha1’ at 39.40%, while the relative conductivities of ‘Zhuyeqi’, ‘9808’, and ‘Taoyuandaye’ after 9 days of drought treatment were 25.42%, 25.96%, and 31.32%, respectively. These findings demonstrate that the drought tolerance of ‘Zhuyeqi’, ‘Taoyuandaye’, and ‘9808’ is significantly higher than that of ‘Bixiangzao’ and ‘Baojing Huangjincha1’, which aligns with their phenotypic results (Fig. S4B).

Our findings indicate that the tea polyphenol content in drought-resistant cultivars, specifically ‘9808’, ‘Taoyuandaye’, and ‘Zhuyeqi’, increased significantly with prolonged drought stress. Notably, the tea polyphenol content in ‘9808’ was significantly higher than in the other tea plant cultivars (*P* < 0.05). In contrast, the tea polyphenol content in non-drought-resistant cultivars exhibited a significant increase on the 3^rd^ and 6^th^ days of drought, followed by a marked decrease after peaking on the 6^th^ day (*P* < 0.05) (Fig. S4C). In summary, the variations in tea polyphenol content are closely associated with the drought resistance of tea plants.

### Effects of exogenous tea polyphenols on root phenotypes and leaf physiological and biochemical changes of tea plants under drought

**Figure 1:**
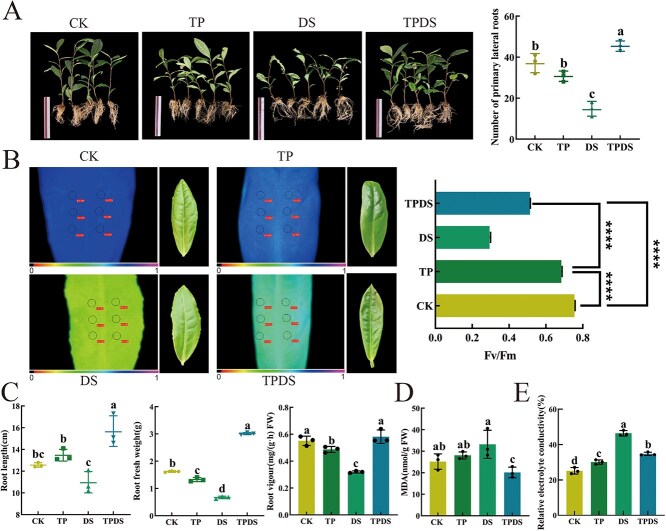
Phenotypic changes in tea plant root systems. (A) Root morphology and the number of white, absorbing roots across four treatment conditions. (B) Chlorophyll fluorescence imaging of tea leaves under four treatment conditions. (C) Changes in root length, fresh weight, and root vitality of tea plants under four treatments. (D) Changes in malondialdehyde (MDA) content in tea plant roots. (E) Changes in relative conductivity of tea plants under four treatments. Three biological replicates were established. Treatment details: CK group received 100 mL of water every 5 days; TP group received 100 mL of tea polyphenols (100 mg/L) every 5 days; DS group received 100 mL of water every 15 days; TPDS group received 100 mL of tea polyphenols (100 mg/L) every 15 days. Statistical significance was determined using one-way ANOVA. Different letters indicate significant differences (*P* < 0.05), and error bars represent the standard deviation (± SD). Statistical significance was also assessed using Student’s *t*-test (^****^P < 0.0001).

To investigate the role of tea polyphenols in the response of tea plants to drought stress, tea polyphenols were externally applied to tea plant seedlings to observe their effects on the plants. In this study, we found that the number of white, absorbing roots in the tea plants of the drought group (TPDS group), which received 100 mL of tea polyphenols at a concentration of 100 mg/L every 15 days, was significantly higher than that of the other groups (*P* < 0.05). In contrast, the tea plants in the drought group (DS group) watered with 100 mL every 15 days exhibited the least white absorptive roots (Fig. 1A). Additionally, the Fv/Fm ratio in the DS group was the lowest, whereas the TPDS group exhibited a significantly higher Fv/Fm ratio compared to the DS group (*P* < 0.05) (Fig. 1B). The root length and fresh weight in the TPDS group were also significantly greater than those in the other groups (*P* < 0.05), measuring 15.7 ± 1.40 cm and 3.0 ± 0.04 g, respectively. In contrast, the DS group exhibited the shortest root length and lowest fresh weight, at 10.9 ± 1.00 cm and 0.67 ± 0.05 g, respectively (Fig. 1C). Furthermore, the TPDS group demonstrated reductions in relative conductivity and malondialdehyde (MDA) content by 14.29% and 12.26%, respectively, compared to the DS group (Fig. 1D and E). Root activity in the DS group was also 29% lower than in the TPDS group (Fig. 1C). These findings suggest that the exogenous application of tea polyphenols can enhance the growth and development of tea plant roots while mitigating the damage caused by drought stress.

The exogenous application of tea polyphenols also had a significant impact on the biochemical composition of tea plant leaves under drought stress. The results showed that gallic acid content was highest in the TPDS group, followed by the DS group, with no significant differences observed between the CK (which received 100 mL of watering once every 5 days) and TP groups (which received 100 mL of tea polyphenols at a concentration of 100 mg/L every 5 days). Theobromine and caffeine, both xanthine alkaloids, were highest in the DS group, with concentrations of 2.071 ± 0.072 mg/g and 42.739 ± 0.325 mg/g, respectively. Theophylline levels did not differ significantly among the four treatments. The concentrations of epigallocatechin (EGC), epigallocatechin gallate (EGCG), and epicatechin gallate (ECG) were significantly higher in the TPDS group than in the other groups (*P* < 0.05). Conversely, the DS group exhibited significantly elevated levels of free amino acids compared to the other groups (*P* < 0.05), while flavonoid content peaked in the CK group and was lowest in the DS group. Soluble sugar content was highest in the DS group and lowest in the CK group (Table 1). These results indicate that the exogenous application of tea polyphenols under drought stress can enhance catechin levels while reducing the accumulation of free amino acids, flavonoids, soluble sugars, theobromine, and caffeine.

**Table 1:** Analysis table of main biochemical components of tea plant.*^a^*

mg/g(DW)	CK	TP	DS	TPDS
Theobromine	0.539 ± 0.017d	1.089 ± 0.023b	2.071 ± 0.072a	0.680 ± 0.015c
Gallic acid	1.705 ± 0.004c	1.617 ± 0.057c	3.126 ± 0.081b	4.161 ± 0.134a
Theophylline	0.146 ± 0.097a	0.126 ± 0.029a	0.209 ± 0.012a	0.182 ± 0.029a
CAF	27.598 ± 0.451d	35.213 ± 0.598c	42.739 ± 0.325a	41.043 ± 0.276b
EGC	33.185 ± 0.376c	30.693 ± 0.336d	40.824 ± 0.378b	45.829 ± 0.376a
DL-C	8.000 ± 0.313a	5.501 ± 0.355c	4.731 ± 0.298d	6.309 ± 0.299b
EC	10.911 ± 0.301a	7.822 ± 0.298c	8.778 ± 0.582b	10.505 ± 0.321a
EGCG	49.605 ± 1.416d	62.602 ± 1.539c	83.461 ± 2.067b	107.337 ± 2.059a
GCG	11.728 ± 0.374a	8.591 ± 0.577b	11.772 ± 0.922a	10.769 ± 0.605a
ECG	14.704 ± 0.571c	15.514 ± 0.841c	17.720 ± 1.010b	25.349 ± 0.483a
Tea polyphenol	145.922 ± 12.503d	166.416 ± 2.511c	196.027 ± 6.928b	246.259 ± 2.801a
Free amino acid	30.350 ± 0.758c	27.512 ± 0.633d	35.377 ± 1.035a	32.364 ± 0.218b
Flavone	10.878 ± 0.185a	9.384 ± 1.028b	5.115 ± 0.282d	7.841 ± 0.184c
Soluble sugar	45.046 ± 0.589d	57.749 ± 0.389c	107.621 ± 5.753a	66.712 ± 5.209b

^a^ Note: Statistical significance was determined using a one-way analysis of variance (ANOVA). Different letters denote statistically significant differences (*P* < 0.05). Abbreviations: CAF, caffeine; C, catechin; EGC, (−)-epigallocatechin; EC, epicatechin; EGCG, (−)-epigallocatechin gallate; ECG, (−)-epicatechin gallate; GCG, (−)-gallocatechin gallate. Treatment details: CK group received 100 mL of water every 5 days; TP group received 100 mL of tea polyphenols (100 mg/L) every 5 days; DS group received 100 mL of water every 15 days; TPDS group received 100 mL of tea polyphenols (100 mg/L) every 15 days.

### Effects of exogenous tea polyphenols on ultrastructural changes and enzyme activities of tea plant roots

**Figure 2:**
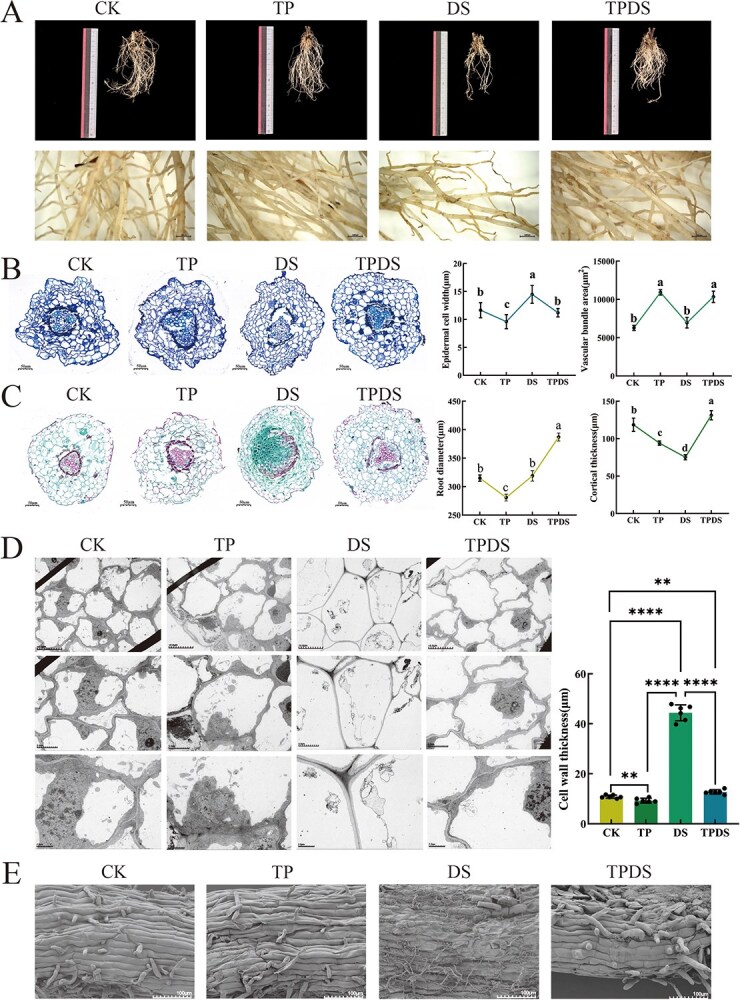
Microscopic phenotypic changes in tea plant roots under various treatments. (A) Differences in root phenotypes among the four treatments (ruler = 1000 μm). (B) Microscopic observation of stained sections of the primary root structure, highlighting variations in epidermal cell thickness and vascular bundle area (ruler = 50 μm). (C) Microscopic observation of stained sections of the primary root structure, showing differences in root diameter and cortical parenchyma cell thickness (ruler = 50 μm). (D) Transmission electron microscopy images of root cells, illustrating differences in cell wall thickness. (The picture rulers from top to bottom are: 10 μm, 5 μm, 2 μm) (The cell wall thickness data of the four treatments are all from the transmission electron microscope results of ruler = 2.0 μm.) (E) Scanning electron microscopy images of root surface morphology (ruler = 100 μm). Treatment details: CK group received 100 mL of water every 5 days; TP group received 100 mL of tea polyphenols (100 mg/L) every 5 days; DS group received 100 mL of water every 15 days; TPDS group received 100 mL of tea polyphenols (100 mg/L) every 15 days. Statistical significance was determined using a one-way analysis of variance (ANOVA). Different letters indicate statistically significant differences (*P* < 0.05). Error bars represent means ± SD (Student’s *t*-test, ^**^P < 0.01, ^****^P < 0.0001).

We conducted some experiments to verify how tea polyphenols affect tea plant root growth under drought stress. Detailed examination of tea plant root systems using stereomicroscopy revealed that under drought conditions, the number of root systems was limited, with the white absorbing root tips appearing shriveled and yellow-brown. In contrast, the TPDS group exhibited better root growth (Fig. 2A). Toluidine blue staining showed that epidermal cell thickness in the DS group was significantly greater than in the other groups (*P* < 0.05). However, no significant difference in epidermal cell thickness was observed between the TPDS and CK groups. The cortical parenchyma cells in the TPDS group appeared approximately round, whereas the DS group showed shrinkage and tearing of endothelial cells, likely due to water loss. There was no significant difference in vascular bundle area between the TP and TPDS groups, while the DS group exhibited a significantly smaller vascular bundle area than the TPDS group (*P* < 0.05) (Fig. 2B). Safranin O-fast green staining revealed severe vascular bundle fibrosis in the DS group. The root diameter of the TPDS group was significantly greater than that of the other groups (*P* < 0.05), and the thickness of cortical parenchyma cells in the TPDS group was 12.38% greater than in the DS group (Fig. 2C).

Scanning and transmission electron microscopy revealed high structural integrity in root tip cells in the CK, TP, and TPDS groups, with well-defined nuclei. In contrast, the DS group exhibited degraded root tip cells, with only cytoplasm, cell membrane, and cell walls remaining. The cell walls in the DS group were significantly thicker, increasing by 60.67% compared to the CK group (Fig. 2D). Scanning electron microscopy indicated that the root surface in the DS group appeared dry and rough, while the TPDS group exhibited a robust root surface with clear lines and dense root hairs (Fig. 2E). Overall, these findings suggest that exogenous tea polyphenols can preserve root cell structure, promote root hair development, and increase vascular bundle thickness, enhancing the drought resistance of tea plants.

**Figure 3:**
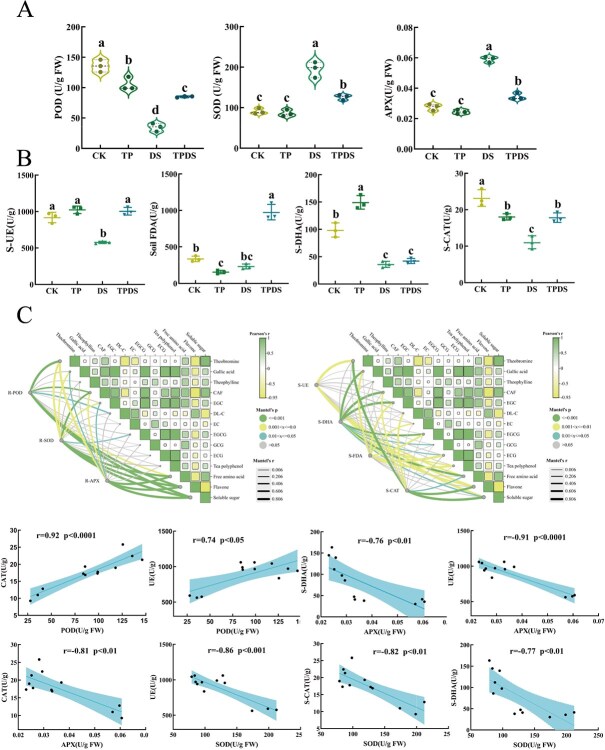
Analysis of enzyme activities in tea plant roots and soil, and their correlations with physiological and biochemical parameters. (A) Activities of root peroxidase (POD), superoxide dismutase (SOD), and ascorbate peroxidase (APX) in tea plant roots under four different treatments. (B) Activities of soil urease (UE), catalase (CAT), hydrolase (FDA), and dehydrogenase (DHA) in the soil under four different treatments. (C) Correlation analysis between root enzyme activities, soil enzyme activities, and physiological indicators of tea plants, as well as a correlation analysis between root enzyme activity and soil enzyme activity. Treatment details: CK group received 100 mL of water every 5 days; TP group received 100 mL of tea polyphenols (100 mg/L) every 5 days; DS group received 100 mL of water every 15 days; TPDS group received 100 mL of tea polyphenols (100 mg/L) every 15 days. Statistical significance was determined using one-way analysis of variance (ANOVA). Different letters indicate statistically significant differences (*P* < 0.05). Error bars represent means ± SD.

Measurements of root enzyme and soil enzyme activities revealed that root peroxidase (POD) activity was significantly lower in the DS group than in the other groups (*P* < 0.05). POD activity in the TPDS group was 42% higher than in the DS group (Fig. 3A). Similar trends were observed for soil urease (S-UE) and catalase (S-CAT) activities, with the DS group showing the lowest activity levels. (Soil- fluorescein diacetate hydrolysis) S-FDA activity was highest in the TPDS group, 61.5% higher than in the DS group (Fig. 3B).

Correlation analysis between root and soil enzyme activities and physiological indicators indicated significant relationships between the activities of (superoxide dismutase) SOD, (aseorbate peroxidase) APX, and free amino acids, flavonoids, theobromine, and soluble sugars. POD activity in tea plant roots was positively correlated with S-CAT and S-UE activities (Fig. 3C). These findings suggest that exogenous tea polyphenols enhance soil enzyme activity under drought conditions, which, in turn, influences root enzyme activity and contributes to improved drought resistance.

### Combined transcriptome and metabolome analysis

**Figure 4:**
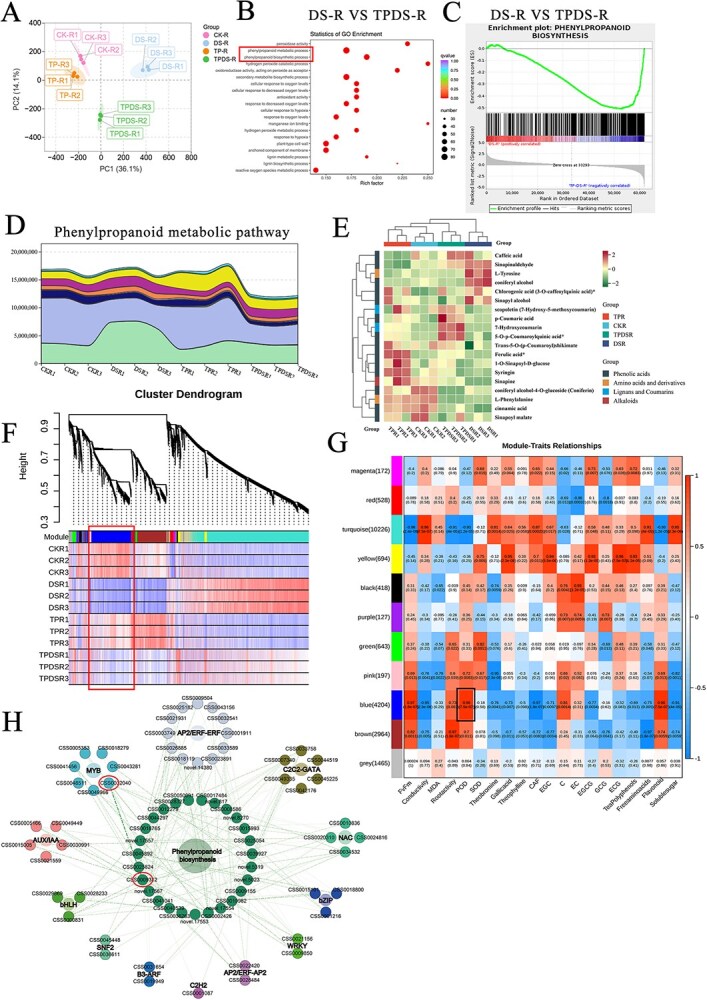
Transcriptome and metabolome analyses of tea plant roots under different treatments. (A) Principal Component Analysis (PCA) score plot depicting gene expression differences in tea plant roots across four treatment groups. (B) Bubble chart illustrating Gene Ontology (GO) enrichment of differentially expressed genes (DEGs) across various pathways in DS group vs TPDS group. (C) Gene Set Enrichment Analysis (GSEA) of phenylpropanoid biosynthesis-related genes, with the highest enrichment observed in the TPDS-R group. (D) Metabolomic analysis of phenylpropanoid biosynthesis pathway (Ko00940) related metabolites under four treatments. Metabolites are divided into 19 types. The upward and downward lines indicate increases or decreases in metabolite content, respectively. (E) Classification of metabolites related to phenylpropanoid biosynthesis pathway (Ko00940) into four main categories: phenolic acids, amino acids and their derivatives, lignans and coumarins, and alkaloids. Changes in their contents across the four treatments are depicted. (F) Modular hierarchical clustering tree from the Weighted Gene Co-expression Network Analysis (WGCNA). (G) Heatmap illustrating the relationship between gene expression modules and sample traits, with numbers indicating correlation coefficients (R) and corresponding *p*-values (in parentheses). (H) Co-expression network of candidate genes within key co-expression modules identified by WGCNA analysis. Treatment details: CK group received 100 mL of water every 5 days; TP group received 100 mL of tea polyphenols (100 mg/L) every 5 days; DS group received 100 mL of water every 15 days; TPDS group received 100 mL of tea polyphenols (100 mg/L) every 15 days.

Principal component analysis (PCA) of transcriptome data revealed distinct gene expression differences between the TPDS-R (tea plant roots received 100 mL of tea polyphenols (100 mg/L) every 15 days) and DS-R groups (tea plant roots received 100 mL of watering once every 15 days) (Fig. 4A). GO (Gene Ontology**)** enrichment showed that differentially expressed genes (DEGs) in the DS group vs the TPDS group were predominantly enriched in the phenylpropanoid biosynthesis and metabolism pathways (Fig. 4B). Furthermore, Gene set enrichment analysis (GSEA) revealed significant upregulation of genes related to the phenylpropanoid pathway in the TPDS-R group (Fig. 4C). In summary, exogenous application of tea polyphenols to tea plants under drought conditions may alleviate drought damage in tea plants by regulating the phenylpropanoid pathway.

**Figure 5:**
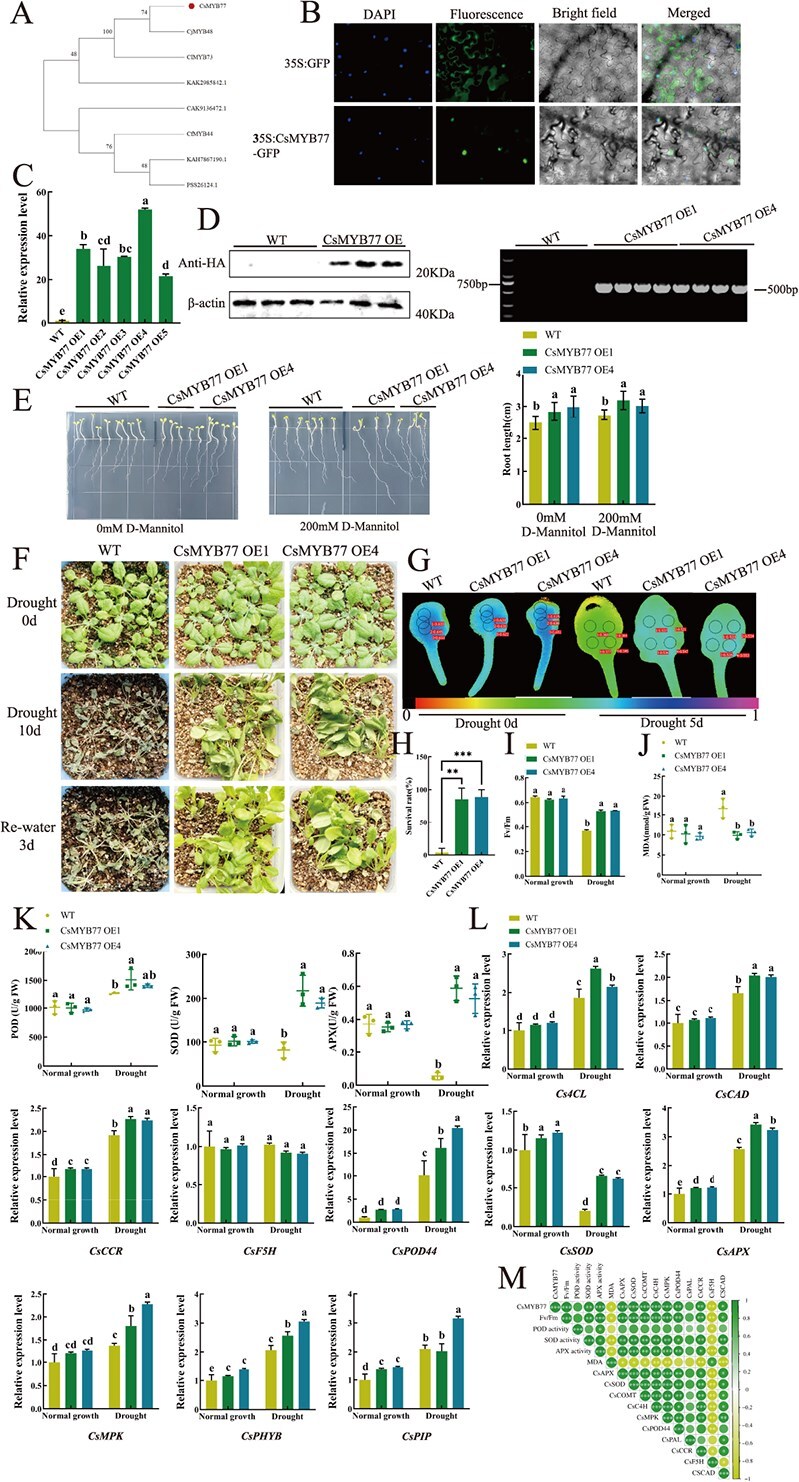
Functional validation of the *CsMYB77* gene in drought resistance. (A) Phylogenetic analysis of *CsMYB77* (highlighted with a dot) in comparison to homologous MYB proteins from various plant species: *Camellia japonica* (*CjMYB48*, WQQ41736.1); *Camellia lanceoleosa* (*ClMYB73*, KAI8028236.1); *Ilex paraguariensis* (unnamed protein product, CAK9136472.1); *Cornus florida* (*CfMYB44*, XP_059652944.1); *Vaccinium darrowii* (hypothetical protein Vadar_030242, KAH7867190.1); *Escallonia rubra* (hypothetical protein RJ640_018708, KAK2985842.1); *Actinidia chinensis* var. *chinensis* (transcription factor-like, PSS26124.1). (B) Subcellular localization of *CsMYB77* in tobacco epidermal cells, scale bar = 10 μm. (C) Quantitative real-time PCR (RT-qPCR) analysis of *CsMYB77* gene expression in transgenic Arabidopsis lines (OE1, OE2, OE3, OE4, OE5) and wild-type (WT) plants. (D) Western blot (WB) analysis of *CsMYB77* protein in transgenic lines and WT, with the protein detected at 23.8 kDa and β-actin at 42 kDa as a loading control. DNA verification results for transgenic lines OE1 and OE4, and WT. (E) Root phenotype comparison between transgenic lines (OE1, OE4) and WT under control (0 mM) and drought-mimicking (200 mM mannitol) conditions, including root length measurements. (F) Phenotypic changes in in transgenic and WT plants (G) Performance of *CsMYB77*-overexpressing *Arabidopsis thaliana* under drought stress: chlorophyll fluorescence imaging on days 0 and 5 of drought stress. (H) Survival rate in transgenic and WT plants under drought stress. (I) Change of Fv/Fm in transgenic and WT plants. (J) Malondialdehyde (MDA) content in transgenic and WT plants. (K) Changes in the activities of peroxidase (POD), superoxide dismutase (SOD), and ascorbate peroxidase (APX) in transgenic and WT lines under normal and drought stress conditions. (L) RT-qPCR detection of phenylpropanoid biosynthesis-related and enzyme activity-related gene expression. (M) Correlation analysis between *CsMYB77* relative expression levels and both enzyme activity and phenylpropanoid biosynthesis-related gene expression in *CsMYB77*-overexpressing Arabidopsis lines. Statistical significance was determined by one-way analysis of variance. Different letters indicate significant differences (*P* < 0.05). Error bars represent ± SD (Student’s *t*-test: ^**^P < 0.01, ^***^P < 0.001).

Tea plant roots’ metabolites were analyzed in the phenylpropanoid pathway (Ko00940 pathway). The results indicated no significant changes in the relative content of metabolites in the CK-R group (which received 100 mL of water every 5 days) and the DS-R group. In contrast, the TP-R group (tea plant roots received 100 mL of tea polyphenols (100 mg/L) every 5 days) exhibited a peak accumulation of metabolites. At the same time, the relative content significantly decreased in the TPDS-R group (Fig. 4D). Additionally, the metabolites were clustered and classified, revealing that the main components included phenolic acids, amino acids and their derivatives, lignans, coumarins, and alkaloids. Among these, phenolic acids represented the most abundant group, while alkaloids were the least prevalent. Notably, the TPDS-R group demonstrated a higher abundance of lignans and coumarins than the other groups, which exhibited minimal levels of these compounds. The DS-R group, in contrast, showed elevated levels of specific phenolic acids, such as sinapinaldehyde and coniferyl alcohol. Meanwhile, ferulic acid, 1-O-sinapoyl-D-glucose, and syringin were more prevalent in the TP-R group (Fig. 4E).

Weighted gene co-expression network analysis (WGCNA) was performed on the transcriptome data to identify key gene modules associated with drought resistance. A threshold of 0.25 was employed to combine similar modules, resulting in the identification of 11 distinct gene modules. These modules were named based on color coding: magenta (172 genes), red (528 genes), turquoise (10 226 genes), yellow (694 genes), black (418 genes), purple (127 genes), green (643 genes), pink (197 genes), blue (4204 genes), brown (2964 genes), and gray (1465 genes). Significant differences in gene expression were observed in the blue module, which demonstrated higher expression in the CK-R and TP-R groups and reduced expression in the DS-R group. The TPDS-R group, however, exhibited significantly increased expression levels (Fig. 4F).

A module-trait relationship heatmap revealed strong positive correlations between module eigenvalues and specific traits. Notably, the blue module displayed a highly significant correlation with peroxidase (POD) activity, yielding a correlation coefficient of 0.96 (Fig. 4G). This suggests that genes within the blue module play a critical role in scavenging reactive oxygen species during drought stress in tea plants, making this module a key candidate for further investigation.

Within the blue module, co-expression network analysis of genes involved in the phenylpropanoid biosynthesis pathway identified 27 genes related to this pathway. Among these, 12 critical regulatory genes were identified, including transcription factors from multiple families: 7 MYB, 12 AP2/ERF-ERF, 6 C2C2-GATA, 4 NAC, 3 bZIP, 2 WRKY, and 5 AUX/IAA (Fig. 4H). Notably, *MYB77* (CSS0032040) exhibited a strong positive correlation with *POD44* (CSS0009312), with a correlation coefficient of 0.9. Based on these results, we focused on the genes *MYB77* and *POD44*, which may regulate the phenylpropanoid pathway and contribute to drought resistance in tea plant roots.

### Expression pattern and subcellular localization of *CsMYB77*

The expression pattern of *CsMYB77* across various tea plant tissues revealed its highest expression in roots, followed by buds, with minimal expression in flowers (Fig. S5). Sequence analysis of *CsMYB77* identified two SANT/MYB conserved domains, classifying it as an R2R3-MYB transcription factor (Fig. S6). Phylogenetic analysis indicated a high level of sequence homology between *CsMYB77* and other R2R3-MYB transcription factors across multiple plant species, with the closest relationship observed with *Camellia japonica* (Fig. 5A).

To determine the subcellular localization of *CsMYB77*, a GFP-tagged *CsMYB77* construct was transiently expressed in tobacco epidermal cells. Fluorescence microscopy revealed that *CsMYB77* was localized in the nucleus, confirming its role as a transcription factor (Fig. 5B).

### Overexpression of *CsMYB77* enhances drought tolerance in *Arabidopsis thaliana*

To investigate the role of *CsMYB77* in drought stress, we generated *Arabidopsis thaliana* lines overexpressing *CsMYB77*. Western blot analysis confirmed the presence of the *CsMYB77* protein in transgenic lines. We selected two overexpression lines (OE1 and OE4) for further drought tolerance experiments based on RT-qPCR validation of *CsMYB77* transcript levels (Fig. 5C and D). Root length measurements on Murashige and Skoog (MS) medium supplemented with 200 mM mannitol, simulating drought conditions, revealed that transgenic *CsMYB77* OE1 and OE4 lines exhibited significantly longer roots compared to wild-type (WT) plants (*P* < 0.05) (Fig. 5E).

Under normal growth conditions, there were no significant differences in vegetative and reproductive growth between transgenic and wild type(WT)plants. However, after 10 days of drought stress, 85.2% and 88.9% of OE1 and OE4 plants, respectively, survived, compared to only 11% of WT plants (*P* < 0.05) (Fig. 5F and H). Furthermore, the Fv/Fm ratio and MDA content in transgenic lines were significantly improved compared to WT under drought conditions (Fig. 5I and J). These results indicate that *CsMYB77* overexpression enhances drought tolerance in *Arabidopsis thaliana*.

Enzyme activity assays revealed that under drought stress, POD, SOD, and APX activities were significantly higher in transgenic lines than in WT plants (Fig. 5K). Additionally, the expression levels of key genes involved in phenylpropanoid biosynthesis, such as *Cs4CL*, *CsCAD*, and *CsPOD44*, were significantly elevated in the OE lines under drought stress (*P* < 0.05) (Fig. 5L). Correlation analysis confirmed significant positive relationships between *CsMYB77* expression and various stress-related genes, further supporting the role of *CsMYB77* in enhancing drought tolerance (Fig. 5M).

### Inhibition of *CsMYB77* reduces drought tolerance in tea plants

**Figure 6:**
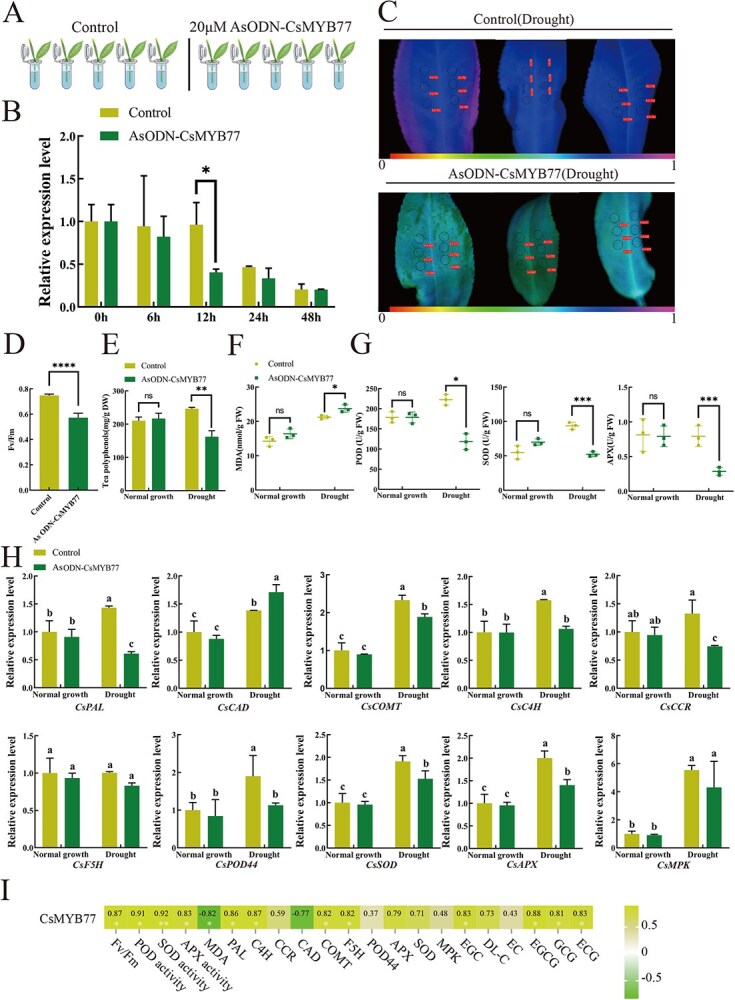
Functional validation of *CsMYB77* in drought resistance through gene silencing. (A) Schematic representation of the experimental procedure for *CsMYB77* gene silencing using antisense oligonucleotides. (B) Relative expression levels of *CsMYB77* following gene silencing at 0, 6, 12, 24, and 48 hours, showing the most pronounced silencing effect at 12 hours. (C) Chlorophyll fluorescence imaging of tea leaves under normal growth and drought stress conditions after *CsMYB77* gene silencing. (D) Comparative analysis of Fv/Fm values, (E) tea polyphenol content, (F) malondialdehyde (MDA) content, (G) peroxidase (POD), superoxide dismutase (SOD), and ascorbate peroxidase (APX) activities between the control group and the *CsMYB77*-silenced group. (H) Quantitative real-time PCR (RT-qPCR) analysis of genes related to phenylpropanoid biosynthesis and enzyme activity following *CsMYB77* gene silencing. (I) Correlation analysis between *CsMYB77* relative expression levels and catechin content, enzyme activities, phenylpropanoid biosynthesis, and other related genes after silencing. Statistical significance was determined using a one-way analysis of variance. Different letters indicate statistically significant differences (*P* < 0.05). Error bars represent ± SD (Student’s *t*-test, ^*^P < 0.05, ^**^P < 0.01, ^***^P < 0.001, ^****^P < 0.0001).

To further assess the function of *CsMYB77* under drought stress, we designed antisense oligonucleotides (AsODNs) to inhibit *CsMYB77* expression in tea plants (Fig. 6A). The silencing of *CsMYB77* significantly reduced drought tolerance, as evidenced by decreased Fv/Fm ratios and increased MDA content in treated plants compared to controls (Fig. 6B–D and F). Additionally, the activities of key antioxidant enzymes (POD, SOD, and APX) were significantly reduced in silenced plants under drought stress (*P* < 0.05) (Fig. 6G).

Analysis of catechin content revealed that silencing *CsMYB77* led to significant reductions in tea polyphenols (*P* < 0.05) (Fig. 6E) and catechin components under drought conditions (Table S3). Furthermore, the expression of phenylpropanoid biosynthesis genes, such as *CsPAL*, *CsCOMT*, and *CsPOD44*, was significantly downregulated following *CsMYB77* silencing (*P* < 0.05) (Fig. 6H and I). These findings demonstrate that the inhibition of *CsMYB77* compromises the drought resistance of tea plants.

### *CsMYB77* binds to the *CsPOD44* promoter and activates its transcription

**Figure 7:**
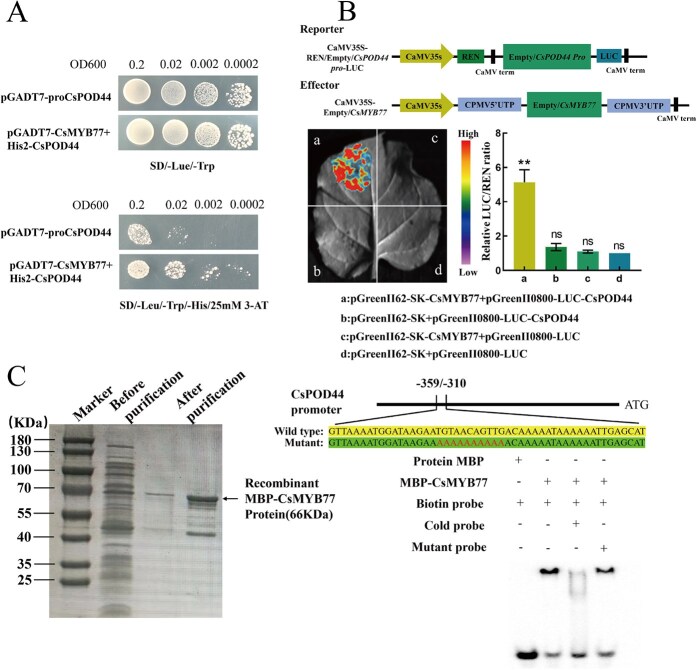
Verification of *CsMYB77* binding to *CsPOD44*. (A) Yeast one-hybrid (Y1H) assay results demonstrating the interaction between *CsMYB77* and the promoter region of *CsPOD44*. (B) Dual-luciferase assay (DLA) and luciferase complementation imaging (LCI) results, confirming the regulatory relationship between *CsMYB77* and *CsPOD44*. (C) Electrophoretic mobility shift assay (EMSA) results for *CsMYB77* binding to the *CsPOD44* promoter. From left to right: MBP-labeled probe with biotin-labeled probe; MBP-CsMYB77 with biotin-labeled probe; MBP-CsMYB77 with biotin-labeled probe and cold competitor probe; and MBP-CsMYB77 with biotin-labeled probe and MBP-labeled mutant probe. Statistical significance was determined using a one-way analysis of variance, with error bars representing ± SD.

To validate the interaction between *CsMYB77* and *CsPOD44*, yeast one-hybrid (Y1H) assays were performed, confirming that *CsMYB77* binds to the *CsPOD44* promoter (Fig. 7A). Luciferase complementation imaging (LCI) and dual-luciferase assays (DLA) further confirmed that *CsMYB77* promotes the transcription of *CsPOD44*, as indicated by a significant increase in fluorescence signals in co-transfected tobacco leaves (*P* < 0.05) (Fig. 7B).

Electrophoretic mobility shift assays (EMSA) showed that *CsMYB77* binds specifically to the promoter region of *CsPOD44* (Fig. 7C). These results demonstrate that *CsMYB77* positively regulates *CsPOD44* expression, contributing to the regulation of the phenylpropanoid biosynthesis pathway and enhancing drought tolerance in tea plants.

## Discussion

### Differences in the metabolism of phenylpropanoid and flavonoids in different tea cultivars lead to differences in their drought resistance

Drought stress is recognized as one of the most detrimental environmental factors affecting plant growth and productivity [25]. In response to drought, plants undergo various morphological, physiological, and biochemical changes. The phenylpropanoid/flavonoid pathway is crucial in plant growth and development. Different tea plant cultivars exhibit specific differences in their metabolic pathways, primarily due to variations in genetic composition and the regulation of these metabolic pathways [26]. Additionally, varying expression levels of enzyme-related genes within the phenylpropanoid/flavonoid metabolic pathway contribute to these differences. Following drought stress, the relative expression of genes involved in the phenylpropanoid/flavonoid pathway, such as *CsPAL*, *CsC4H*, and *CsCCR*, significantly increased (*P* < 0.05), thereby accelerating the metabolism of this pathway (Fig. 6H). This metabolic acceleration results in varying changes in the content of tea polyphenols and catechins across different tea plant cultivars, leading to diverse drought tolerance responses under drought stress. Tea polyphenols possess intense antioxidant activity and are vital in mitigating oxidative damage caused by reactive oxygen species [27]. Notably, the tea polyphenol content in drought-resistant tea plant cultivars was significantly higher compared to non-drought-resistant cultivars (*P* < 0.05) (Fig. S4). This indicates that tea polyphenols have a positive regulatory role in drought stress, indicating that tea plant cultivars with higher tea polyphenol content may exhibit greater drought tolerance than those with lower levels.

In this study, an external application was utilized to assess the impact of tea polyphenols on drought stress in the roots of tea plants. The exogenous application of tea polyphenols under drought conditions significantly enhanced root growth, as evidenced by increased root length, fresh weight, root vigor (Fig. 1C), and elevated levels of catechins and tea polyphenols in the leaves (Table 1), while simultaneously reducing MDA content (Fig. 1D) (*P* < 0.05). These findings highlight the capacity of exogenously applied tea polyphenols to alleviate drought-induced inhibition of root development in tea plants. Additionally, previous research has indicated that applying exogenous boron under salt stress promotes the development of root systems and restores cellular integrity [28]. Furthermore, compared to the DS group, the activity of POD in the TPDS group was significantly elevated (*P* < 0.05) (Fig. 3A). This suggests that POD may play a crucial role in enhancing drought stress tolerance mediated by tea polyphenols. Consequently, the selective stimulation of antioxidant enzyme activity induced by tea polyphenols may enhance plant resilience to abiotic stress. However, the mechanisms by which signals induced by tea polyphenols regulate antioxidant enzyme activity at the transcriptional level warrant further investigation. Moreover, tea polyphenols promote the accumulation of catechins within the flavonoid biosynthetic pathway, providing valuable insights for further elucidating the mechanisms underlying abiotic stress tolerance induced by tea polyphenols.

**Figure 8:**
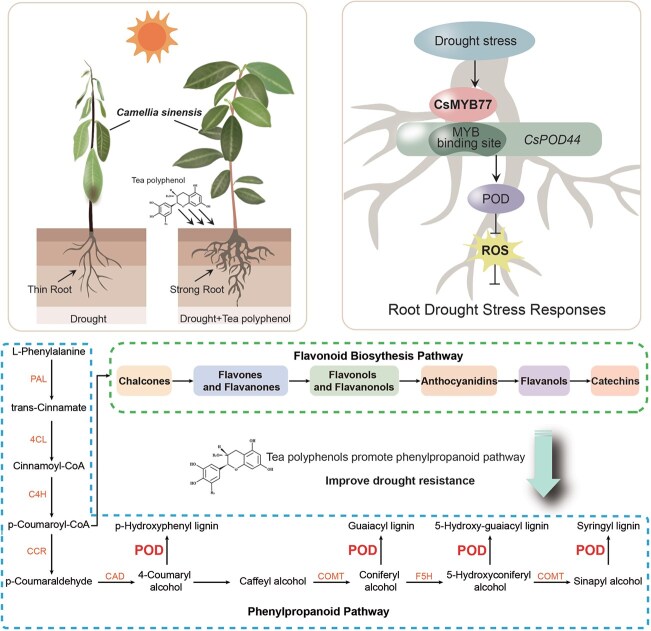
Growth status of tea plants under drought stress with and without the exogenous application of tea polyphenols. The proposed model illustrates the role of *CsMYB77* in directly targeting *CsPOD44* to enhance drought tolerance in tea plants. Tea polyphenols, secondary metabolites in the flavonoid pathway, can activate the phenylpropanoid biosynthetic signaling pathway, thereby increasing peroxidase (POD) activity within this pathway. This elevation in POD activity facilitates the scavenging of excessive reactive oxygen species (ROS), ultimately improving drought tolerance in tea plant roots. Additionally, this process may promote the accumulation of downstream phenylpropanoid products, contributing to ROS elimination. Further experimental verification is required to confirm this mechanism.

The phenylpropanoid metabolism pathway is crucial in plant development and plays an essential role in plant-environment interactions [29]. The genetic regulation of phenylpropanoid and flavonoid biosynthesis, particularly the genes encoding structural enzymes, has been well-characterized in model plants such as *Arabidopsis thaliana* [30–33]. In this study, MYB family transcription factors emerged as key regulators in the phenylpropanoid biosynthesis process. GO enrichment analysis revealed that differentially expressed genes were predominantly enriched in the phenylpropanoid biosynthesis and metabolism pathways (Fig. 4B). Additionally, WGCNA identified *CsMYB77* and *CsPOD44* as pivotal genes involved in this pathway (Fig. 4H). The findings indicate that *CsMYB77* and *CsPOD44* play a crucial regulatory role in the ability of tea polyphenols to induce drought tolerance in tea plants. Tea polyphenols are synthesized through the flavonoid pathway, representing a significant downstream branch of the phenylpropanoid metabolic pathway. Flavonoids are known to contribute to plant responses under drought stress (Fig. 8). Consequently, tea polyphenols may enhance phenylpropanoid metabolism, activate the *CsMYB77* gene associated with this pathway to regulate *CsPOD44*, and increase POD activity within the phenylpropanoid pathway, ultimately improving the drought resistance of tea plant roots. This conclusion is consistent with previous research demonstrating that phenylpropanoid and flavonoid metabolic pathways respond to drought stress in soybeans [34] and sustain high POD activity to counteract excess ROS accumulation [35].

### Tea polyphenols improve tea plant root drought resistance by regulating *CsMYB77*-*CsPOD44* module

MYB transcription factors are extensively studied for their role in abiotic stress responses across various plant species, including *Arabidopsis thaliana*, *Gossypium hirsutum*, and *Malus pumila* [36–38]. These transcription factors play pivotal roles in mediating responses to environmental stresses. For instance, under drought conditions, the cotton gene *GhMYB36* is highly inducible, with gene silencing resulting in increased drought sensitivity and overexpression enhancing drought tolerance [39]. In this study, *CsMYB77*, a transcription factor isolated from the tea plant root system, was found to be highly homologous to *Camellia japonica* (Fig. 5A). Silencing *CsMYB77* through antisense oligonucleotides significantly increased drought sensitivity in tea plants (*P* < 0.05) (Fig. 6C, D, and F). In contrast, overexpression of *CsMYB77* in *Arabidopsis thaliana* resulted in increased POD activity (*P* < 0.05), reduced MDA content (*p* < 0.05), and elevated expression of *CsPOD44* (*P* < 0.05) (Fig. 5J–L). Compared to wild-type plants, overexpression lines exhibited significantly improved survival rates and reduced drought-induced damage (*P* < 0.05) (Fig. 5H). Additionally, we observed significant upregulation of the auxin gene *CsIAA8* and the photosynthetic system gene *CsHY5* in the roots of overexpression lines (*P* < 0.05) (Fig. S7), suggesting that *CsMYB77* may regulate hormone-related and light-responsive genes involved in root growth. Previous studies have shown that interactions between the ABA receptor *PYL8* and *MYB77* enhance auxin-related transcriptional activity, promoting lateral root development [40]. The Arabidopsis transcription factor *MYB77* regulates auxin signaling [41], and its interaction with UV-B photoreceptor UVR8 modulates auxin response and lateral root growth [42].

Tea polyphenols exhibit strong antioxidant activity and can modulate the expression of abiotic stress-related genes, thereby influencing the stress resistance of plants. Research has demonstrated that jasmonic acid positively regulates the accumulation of cold-induced flavanols through the *JAZ*-*MYB12* module, enhancing the cold resistance of plants [43]. Additionally, ABA improves drought resistance by regulating genes associated with flavonoid metabolism in pigeon peas [44]. Furthermore, 5-aminolevulinic acid enhances cold tolerance by modulating the *SlMYB4*/*SlMYB88*-*SlGSTU43* module, which scavenges reactive oxygen species in tomatoes [45]. In this study, techniques such as DLA, LCI, Y1H, and EMSA revealed that *CsMYB77* can positively regulate the expression of *CsPOD44*. It was shown that tea polyphenols effectively scavenge excessive reactive oxygen species in tea plants, thereby improving their drought resistance by regulating the *CsMYB77*-*CsPOD44* module within the phenylpropanoid pathway (Fig. 7). This research is the first to demonstrate that tea polyphenols can mitigate drought-induced damage to tea plant roots and further elucidates that the signaling mechanism by which tea polyphenols induce drought tolerance in tea plants involves the regulation of *CsPOD44* transcriptional expression through *CsMYB77*. Moreover, it was found that exogenous tea polyphenols may serve as effective anti-stress auxiliaries to enhance the stress resistance of tea plants. Consequently, this study offers novel insights into drought management in tea gardens, the selection of drought-resistant cultivars, and the potential application of tea polyphenols as stress-resistance auxiliaries.

In summary, this study investigates the molecular mechanisms by which tea polyphenols mediate the response of tea plants to the severe drought conditions observed in recent years. The research reveals that variations in the metabolism of phenylpropanoids and flavonoids among different tea plant cultivars contribute to their distinct drought resistance capabilities. It was determined that tea polyphenols play a significant role in enhancing the drought resistance of tea plants. Exogenous application experiments were conducted to validate the effects of tea polyphenols further, demonstrating that these compounds can mitigate drought-induced damage to the root systems of tea plants. Through a combined analysis of transcriptomic and metabolomic data, two genes, *CsMYB77* and *CsPOD44*, were identified within the phenylpropanoid pathway. Subsequent experiments involving Arabidopsis overexpression and tea plant antisense oligonucleotides confirmed that *CsMYB77* possesses notable drought-resistance properties. Finally, molecular experiments were performed to elucidate the mechanisms by which tea polyphenols respond to drought stress via the *CsMYB77*-*CsPOD44* module in the phenylpropanoid pathway. Consequently, this study offers a comprehensive analysis of how tea polyphenols enhance root resistance in tea plants at the transcriptional level, providing new insights into their role in drought resistance.

## Materials and methods

### Experimental materials

The plant materials utilized in this study were annual tea seedlings derived from cuttings of the drought-resistant ‘9808’ cultivar, which was developed in Anhua County, Yiyang City, Hunan Province, China. The seedlings were cultivated in the tea plantation of Hunan Agricultural University (113°08′ E; 28°18′ N). In December 2020, 540 seedlings were transplanted into polyethylene pots, with each pot containing three plants and 7 kg of soil, resulting in a total of 180 pots. The average seedling height was 28 cm. The dimensions of the pots were as follows: 26.5 cm outer diameter at the upper opening, 23 cm inner diameter at the upper opening, 18 cm diameter at the bottom, and 25 cm height. The physicochemical properties of the soil were as follows: total nitrogen content, 0.994 g/kg; hydrolytic nitrogen, 53.8 mg/kg; available phosphorus, 0.930 mg/kg; available potassium, 52.3 mg/kg; organic matter, 16.8 g/kg; and pH 4.63. After transplantation, the potted plants were placed in a transparent greenhouse with adequate roof coverage and air circulation. The experimental treatments were initiated after a six-month acclimatization period [24].

### Experimental treatment

Tea polyphenols used in this study were purchased from Hunan Aijia Biotechnology Co., Ltd., China. The first drought treatment commenced on June 10, 2022, and lasted for 9 days. Four treatment groups were established: (1) the control group (CK) without drought stress; (2) the TP group, in which plants were irrigated with 100 mg/L tea polyphenols (TP) without drought stress; (3) the DS group, subjected to drought stress; and (4) the TPDS group, where tea polyphenols were applied prior to drought stress. Based on the preliminary results, the second drought treatment began on July 25, 2022, and extended for 60 days. The same four treatment groups were established, with 100 mL of water or tea polyphenols (100 mg/L) administered at intervals of 5 or 15 days, depending on the treatment. Root length, root number, root fresh weight, and other parameters were recorded. Samples were snap-frozen in liquid nitrogen and stored at −80°C for subsequent analyses, with each parameter measured in triplicate [24].

### Determination of tea plant root phenotypes, physiological indicators and MDA content in *Arabidopsis thaliana*

Root samples from tea plants were carefully washed and analyzed for primary root length, lateral root count, and fresh weight using direct measurement methods and three biological replicates were established. Microscopic images of lateral roots were captured using a Zeiss Axiocam 208 color microscope (Carl Zeiss AG, Germany). Root activity was quantified with a plant root activity detection kit (Coolaber, China), and MDA content in tea plant roots was measured using an assay kit (Item NO: BC0025, Solarbio, China). MDA content in *Arabidopsis thaliana* was determined using the CheKine detection kit (Item NO: KTB1050, Abbkine, China). According to the GB/T8314 2013 standard (China), the ninhydrin colorimetric method was employed to assess free amino acid content changes. The soluble sugar content was measured using the anthrone colorimetric method, as outlined by Redillas et al [46]. The flavonoid content was quantified using a spectrophotometer, following the procedure described by Nguyen et al [47]. The determination of relative conductivity was conducted by the method established by Wang et al [48]. Three biological replicates were established.

### Chlorophyll fluorescence parameters

Leaf samples were dark-adapted at room temperature for 25 minutes, after which the maximum quantum yield of photosystem II (Fv/Fm) was measured using the PAM fluorescence imaging system (WALZ, Germany). Three biological replicates were established.

### Detection of root apex anatomy

Root apex samples were fixed using methanol-acetic acid-glacial acetic acid (70% FAA), stained with safranin O-fast green and toluidine blue, dehydrated, and sealed with neutral gum. Photographic images were captured using a Nikon microscope (Japan) and analyzed using CaseViewer software. For transmission electron microscopy, samples were embedded, polymerized, sectioned, and stained, followed by imaging with a Hitachi transmission electron microscope (Japan). Scanning electron microscopy was performed using a Quorum critical point dryer (UK) and a Hitachi scanning electron microscope (Japan). Three biological replicates were established.

### Plant and soil enzyme activity assays

Plant enzyme activities of SOD, POD, and APX were quantified using specific activity detection kits (Item NO: BC0175, BC0095, and BC0225, respectively) (Solarbio, China). Soil enzyme activities, including catalase, hydrolase, dehydrogenase, and urease, were measured using appropriate activity kits (Item NO: BC0100, BC0480, BC0390, and BC0120, respectively) (Solarbio, China). Three biological replicates were established.

### Liquid chromatograph-mass spectrometer (LC–MS/MS) analysis

Analyses were conducted using a Shimadzu Nexera X2 LC-30 AD system (Kyoto, Japan). Samples were separated on an Acquity BEH C18 column (2.1 × 100 mm, 1.7 μm) (Waters, USA), maintained at 40°C. The gradient utilized 0.1% formylic acid (FA) in water and 0.1% FA in acetonitrile as eluents, with a flow rate of 0.3 mL/min and an injection volume of 1 μL. Three biological replicates were established.

### Transcriptome analysis

Twelve tea root samples from the four experimental treatments were sent to Metware Biotechnology Co., Ltd. (China) for transcriptome sequencing, with three biological replicates per group. Transcriptome data results were available for subsequent analysis (Table S1). Differentially expressed genes (DEGs) were identified using DESeq2 v1.22.1, with adjusted p-values <0.05 and |log_2_FoldChange| ≥1 used as thresholds for significant DEGs. The transcriptome data has been uploaded to the public database NCBI under the accession number PRJNA1193475 and attached in Table S4.

### Metabolomic analysis

The metabolomic profiling of tea plant roots from the four treatments was conducted by Metware Biotechnology Co., Ltd. (China) (Fig. S1). Quantitative analysis of metabolites in the phenylpropanoid biosynthetic pathway (Ko00940) was performed using a self-built MWDB (Metware database). Raw data is attached in Table S5.

### Weighted gene co-expression network analysis (WGCNA)

We filtered the input FPKM expression file and utilized the varfilter function from the R language gene filter package to eliminate genes with low expression across all samples and those with stable expression throughout. The pickSofTthreshold function was employed to determine the optimal soft threshold. The threshold for the square of the scale free topology model fit correlation coefficient was set at 0.85. Based on these results, we generated a scatter plot corresponding to the power values and selected 9 as the most suitable power value (Fig. S2). This selection ensures the gene network adheres to a scale-free network distribution, enhancing its biological relevance. A co-expression network was constructed using the optimal soft threshold, and the genes were categorized into distinct modules, creating a gene clustering tree. Correlation heatmaps were then drawn to illustrate the relationships between modules and trait phenotypes. Additionally, a key module network was constructed in Cytoscape software based on the similarity among genes within key modules, under the conditions of *P* < 0.9, to visualize the interactions between genes in the network. Genes of different modules are attached in Table S6.

### RNA extraction and quantitative real-time PCR (RT-qPCR) analysis

Total RNA was extracted from tea plant roots using the FastPure Universal Plant Total RNA Isolation Kit (Vazyme, China). First-strand cDNA was synthesized using the Evo M-MLV reverse transcription reagent kit (Agbio, China), and RT-qPCR was performed using the BYBR Green Premix Pro Taq HS qPCR kit (Agbio, China) on a QuantStudio 3 system (Thermo Fisher Scientific, USA). Gene expression was normalized using the 2^-ΔΔCT^ method, with three biological replicates per sample (Table S2).

### Subcellular localization of *CsMYB77*

The coding region of *CsMYB77* (CSS0032040) was inserted into the pEAQ-GFP vector to form a GFP-fusion construct. The GV3101 agrobacterium tumefaciens strain, transformed with the pEAQ-*CsMYB77*-GFP construct encoding a green fluorescent protein (with an OD600 of 0.6–0.8), was injected into tobacco plants. The agrobacterium strain carrying the pEAQ-GFP vector of green fluorescent protein was also injected into tobacco as a control. The plants were initially treated under low light conditions at 25°C for 12 hours, then transferred to normal light conditions for 48 hours. GFP signals were detected using a Carl Zeiss upright fluorescence microscope (Germany). Primers are shown in Table S2. Three biological replicates were established.

### Luciferase complementation imaging (LCI) and dual-luciferase assay (DLA)

A 618 bp fragment of *CsMYB77* was cloned into the pGreenII62-SK vector as the effector, while the *CsPOD44* promoter was inserted into the pGreenII0800-LUC vector as the reporter. The GV3101 (pSoup-p19) strain of agrobacterium tumefaciens was transformed and subsequently mixed with strains carrying effector and reporter genes. This mixture was co-transfected into the epidermis of tobacco leaves. The plants were initially grown under low light conditions at 25°C for 12 hours, then transferred to normal light conditions for 2–3 days to facilitate infection and gene expression. For the infection process, fluorescein potassium salt solution was applied to the underside of the tobacco leaves, and the fluorescence signals were detected using the Viber Newton 7.0 Bio plant live imaging system (France). The Dual Luciferase Reporter Gene Assay Kit (TransGen Biotech, China) was also employed to assess dual luciferase activity. Three biological replicates were conducted, and the primers utilized are detailed in Table S2.

### Yeast one-hybrid (Y1H) assay

A fragment of *CsMYB77* was cloned into pGADT7 as the prey vector, while a fragment of *CsPOD44* was inserted into pHis2 as the bait. The constructs were co-transformed into yeast strain Y187, after successfully transforming the yeast, select a fresh single colony (2–3 mm) from each sample and resuspend it in 1 mL of sterile water. Adjust the optical density at 600 nm (OD600) to 0.2, and subsequently dilute it with sterile water to achieve 10-fold, 100-fold, and 1000-fold dilutions (OD600 = 0.2, 0.02, 0.002, 0.0002). From each dilution, take 10 μL and inoculate onto the corresponding SD/−Leu/−Trp and SD/-His/−Leu/−Trp plates containing 3-AT(3-amino-1,2,4-triazole). Incubate these plates at 30°C for 2 to 3 days and observe the recombinant yeast in each group. The growth conditions on plates with the corresponding autoactivated 3-AT concentrations will be used to assess potential interactions. Ensure that three biological replicates are established. Primers are shown in Table S2.

### Electrophoretic mobility shift assay (EMSA)

The coding region of *CsMYB77* was inserted into pMAL-c6T, expressed in E. coli BL21, and induced with IPTG. MBP fusion protein purification was performed using a kit from New England Biolabs (USA). To synthesize a universal probe, select a 50 bp DNA fragment that encompasses the promoter region of the MYB binding site located at the 5′ end of the *CsPOD44* sequence and append an MBP tag to the 5′ end. The cold probe sequence mirrors the ordinary probe but lacks the MBP label at the 5′ end. To synthesize a mutation probe, all MYB site sequences (TGTAACAGTTG) sequences are substituted with A bases, while the remaining sequences remain unchanged from those of the ordinary probes. All probes were synthesized by Beijing Qingke Biotechnology Co., Ltd. (Tsingke, China). The probes and samples were subjected to a binding reaction and electrophoresis in a 6% polyacrylamide gel, utilizing TBE as the electrophoresis buffer. Images were captured using an Invitrogen iBright CL1500 imaging system (Thermo Fisher Scientific, USA). Three biological replicates were established. The biotin-labeled probe sequences utilized are detailed in Table S2.

### *Arabidopsis thaliana* overexpression experiment

A fragment of *CsMYB77* was inserted into the p1300 vector to generate the *35S::CsMYB77-EYFP* construct. This construct was transformed into *Arabidopsis thaliana* (Col-0) (wild type, WT). Collect T1 generation seeds, followed by disinfection and sterilization. Subsequently, inoculate the seeds to grow on a medium containing hygromycin B to screen for positive plants. The identified positive plants were then transplanted into the soil for further cultivation. Obtain the T2 and T3 generation homozygous line OE transgenic Arabidopsis, and screen and identify the expected transgenic plants through DNA verification, Western blot (WB) experiments, and other methods. Select the T3 generation homozygous line OE for subsequent verification and analysis. Three biological replicates were established, and the primers used are listed in Table S2.

### Western blot

Total protein was extracted from *Arabidopsis thaliana* using the Coolaber Plant Protein Extraction Kit (China). After SDS-PAGE, proteins were transferred to a PVDF membrane, and incubation was conducted using the HA Tag Mouse monoclonal antibody (mAb) as the primary antibody (Beyotime, China) and the anti-mouse IgG HRP-conjugated secondary antibody for immunoprecipitation (IP) (Vazyme, China). The Actin (26F7) Mouse mAb for PLANTs (Abmart, China) was an internal control. Immunoreactive bands were detected using the Invitrogen iBright CL1500 system. Three biological replicates were established.

### Antisense oligonucleotide (AsODN) experiments

Antisense oligonucleotides (AsODNs) targeting CsMYB77 were designed using Soligo software. Tea plant buds were treated with 20 μM AsODNs, while a separate group was treated with a sense strand solution (sODN) as a control. Gene silencing was assessed after 6, 24, and 48 hours. The group exhibiting effective gene silencing was selected and placed in an empty 2 ml centrifuge tube to natural drought treatment for 3 hours. In contrast, the control group (CK) was placed in sterile water to facilitate normal growth for the same duration. Subsequently, chlorophyll fluorescence parameters were assessed. Additionally, it is essential to establish three biological replicates for the experiment. The primers used are listed in Table S2.

### Statistical analysis

Data were analyzed using IBM SPSS Statistics 23.0 (IBM, USA), with statistical significance determined by Student’s *t*-tests and Duncan’s multiple range comparisons at *P* < 0.05. Graphs were plotted using GraphPad Prism 9.0, chiplot, OmicShare Tools, and Bioinformatics platforms.

## Supplementary Material

Web_Material_uhaf048

## Data Availability

The data that support the results are provided in this paper and its supplementary files.
