# Supplementary material for: Tea polyphenol mediated CsMYB77 regulation of CsPOD44 to promote tea plant (Camellia sinensis) root drought resistance
Source: Hortic Res. 2025 Feb 18;12(6):uhaf048. doi: 10.1093/hr/uhaf048 (PMC12010877; doi:10.1093/hr/uhaf048)
Supplement: Web_Material_uhaf048 [file web_material_uhaf048.zip › Supplemental figures.docx]

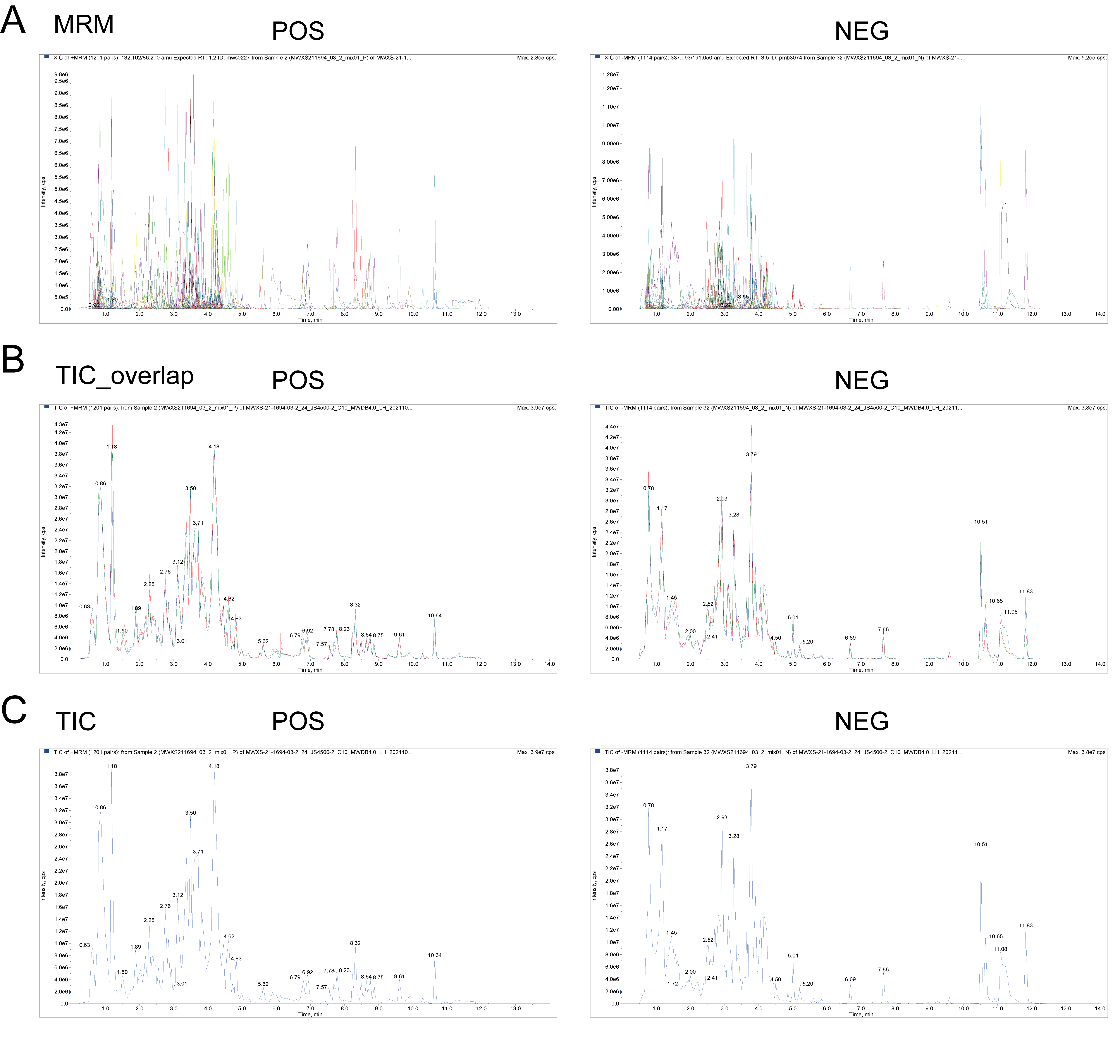
**Figure S1.** Metabolome analysis. (A) MRM detection of multimodal maps. (B) Quality control sample mass spectrometry detection TIC overlay chart. (C) Total ion chromatogram of mass spectrometry analysis of quality control samples.


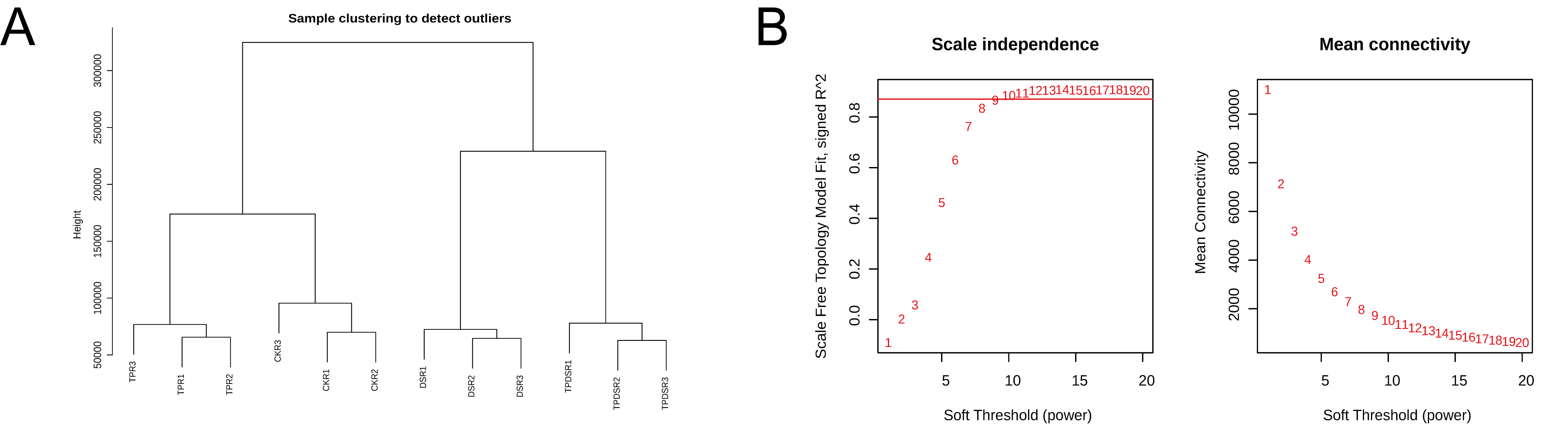


**Figure S2.** WGCNA analysis. (A) Sample clustering tree, you can view the correlation between samples through the sample clustering tree. (B) Power value selection diagram.


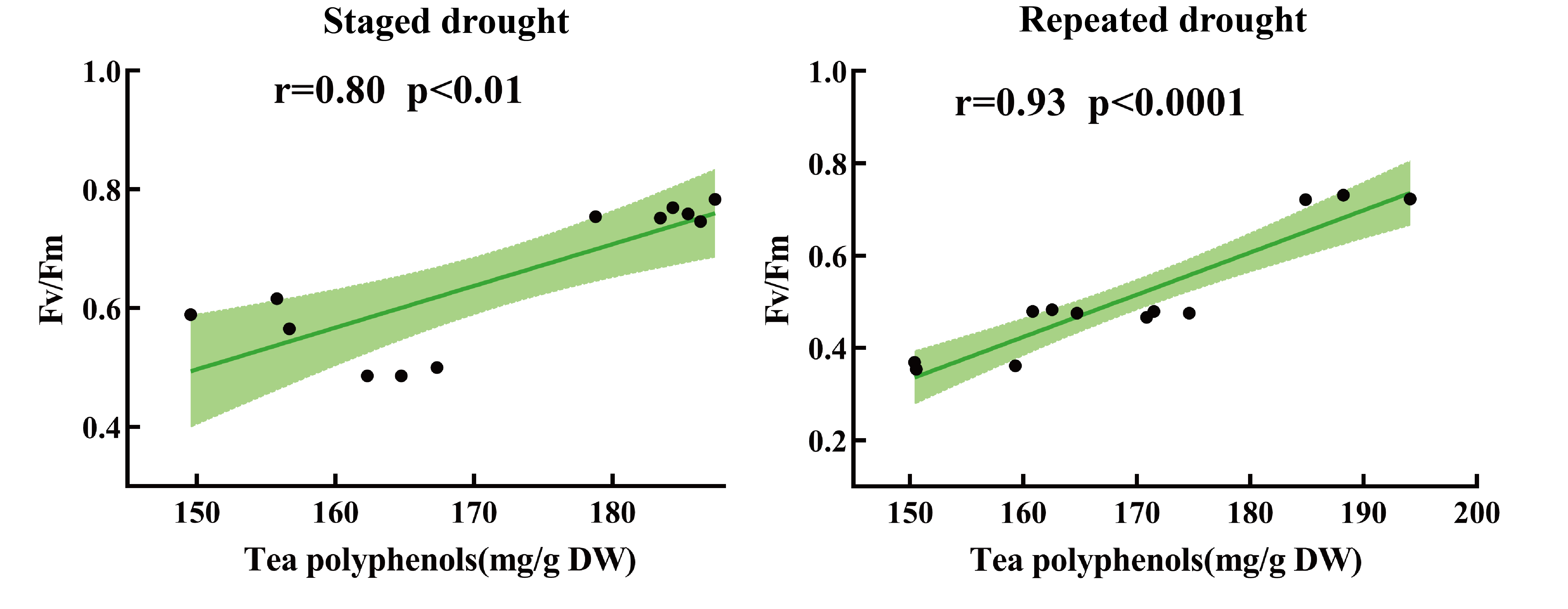


**Figure S3.** Correlation analysis between tea polyphenols content and Fv/Fm under repeated and staged drought conditions. Repeated drought treatments for tea plants are conducted in several stages. The first drought treatment occurs from July 24 to August 5, 2021 (D1), followed by the second treatment from August 15 to August 27 (D2), and the third treatment from September 6 to September 18 (D3), CK (control group). The staged drought includes short-term drought, long-term drought, and rehydration phases. It is important to note that the drought treatment will commence on July 24, 2021. Sampling will take place at four time points: July 24, August 2, August 9, and August 17, corresponding to days 0, 9, 16, and 8 of the rehydration phases, respectively. The samples collected will be designated as CK, DS1, DS2, and RD in chronological order.


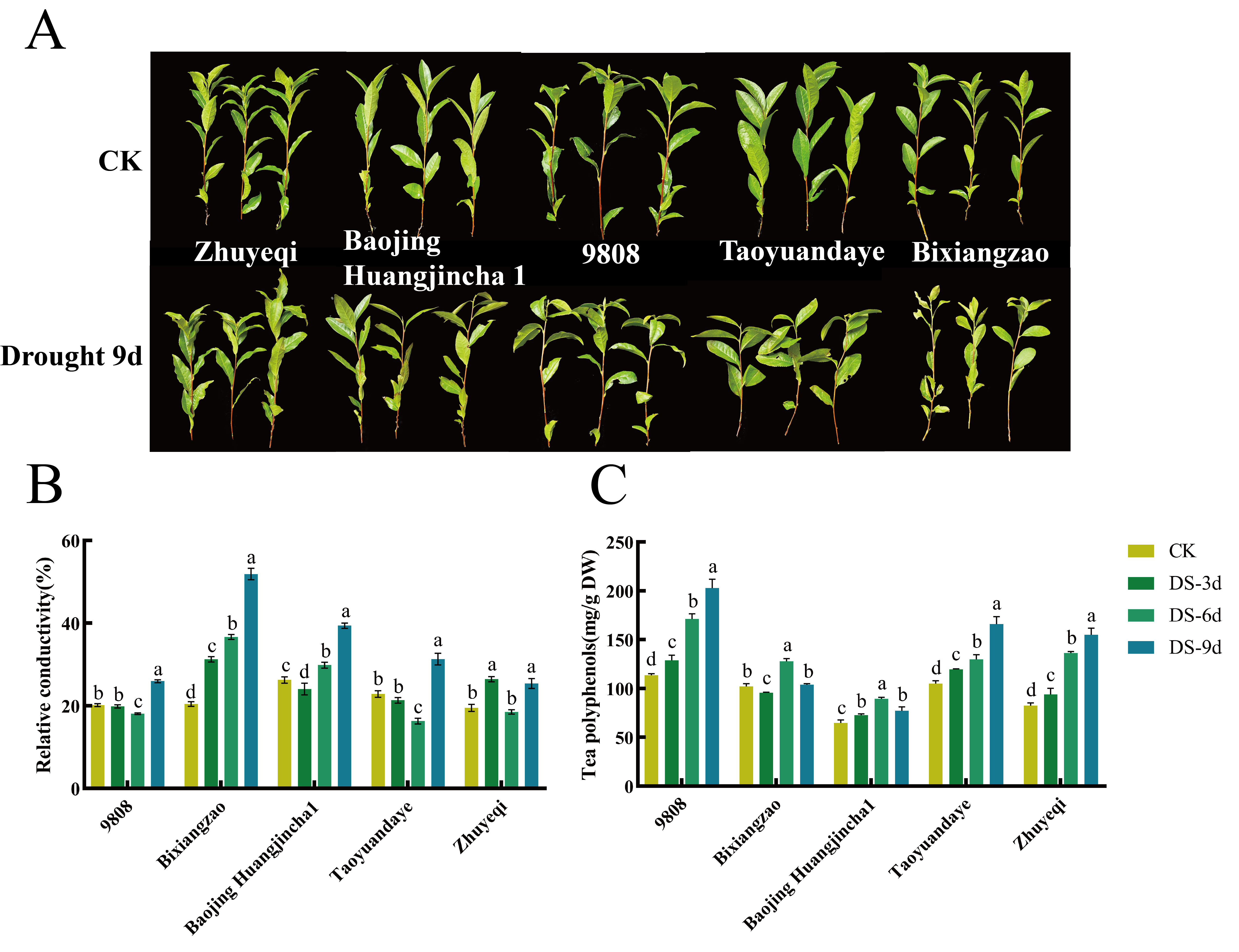


**Figure S4.** Changes in phenotype(A), relative conductivity(B) and tea polyphenol content(C) of different five tea plant cultivars under drought stress. The drought treatment commenced on June 10, 2022, and continued for 9 days. Samples were collected on June 10 (CK), June 13 (DS-3d), June 16 (DS-6d), and June 19 (DS-9d) to measure the relative conductivity and the content of tea polyphenols. Statistical significance was determined using one-way analysis of variance (ANOVA). Different letters indicate statistically significant differences (*p* < 0.05). Error bars represent means ± SD.

**
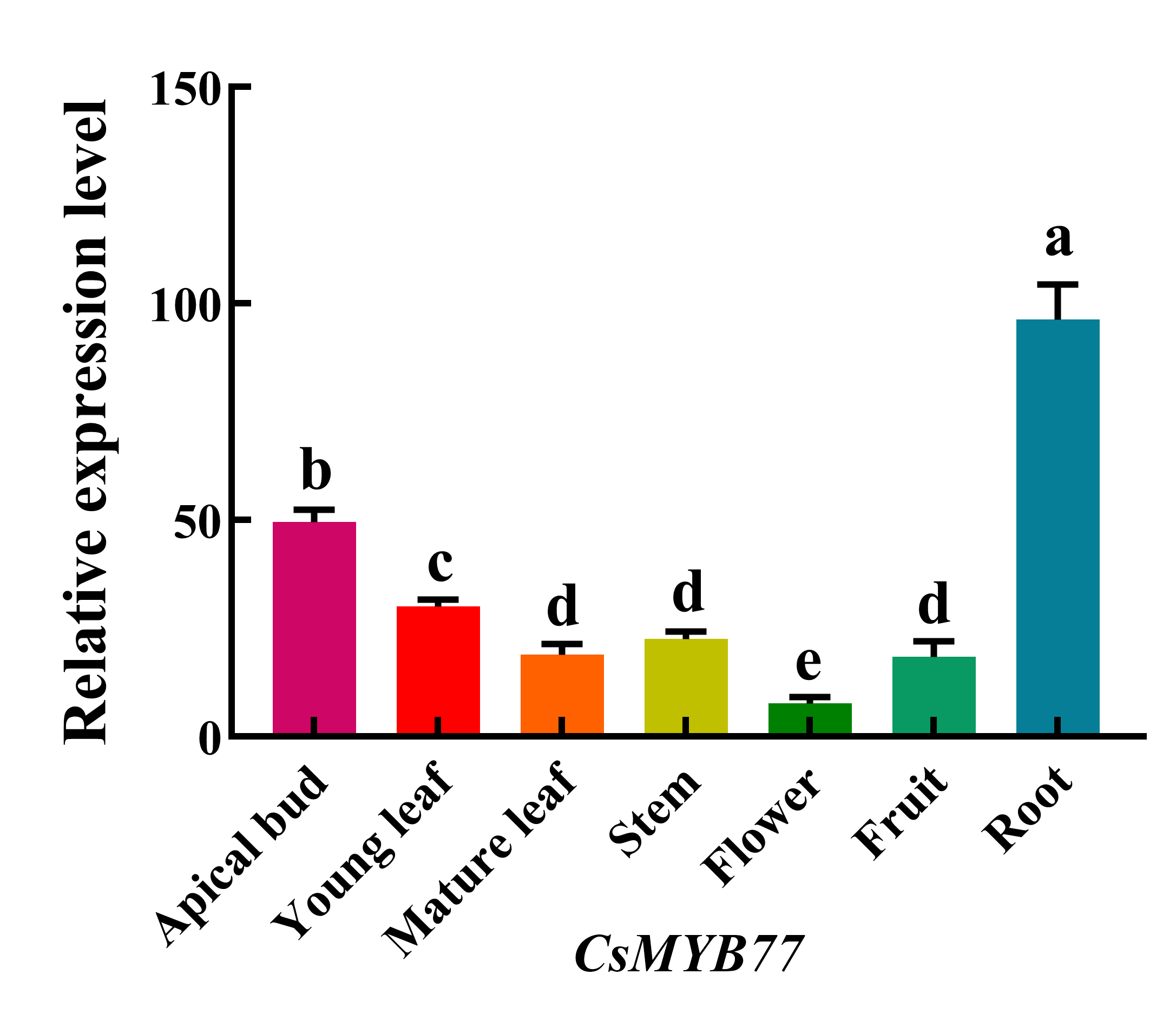
**

**Figure S5.** Relative expression of *CsMYB77* in different tissues of tea plant, as determined by qRT-PCR (*p* < 0.05); the data are presented as the means±standard errors of 3 replicates. Different letters indicate significant differences.


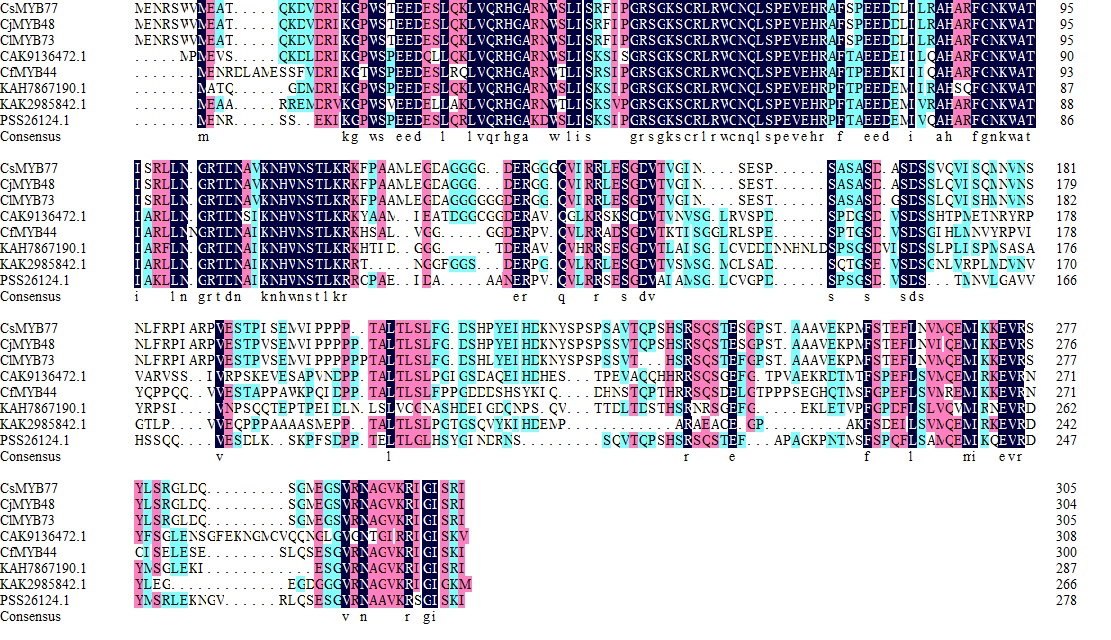


**Figure S6.** Sequence alignment of the *CsMYB77* protein with protein homologs in other plant species. The proteins used in this analysis are as follows: *Camellia japonica*: *CjMYB48* (WQQ41736.1); *Camellia lanceoleosa*: *ClMYB73* (KAI8028236.1); *Ilex paraguariensis:* unnamed protein product (CAK9136472.1); *Cornus florida*: *CfMYB44* (XP_059652944.1); *Vaccinium darrowii*: hypothetical protein Vadar_030242 (KAH7867190.1); *Escallonia rubra*: hypothetical protein RJ640_018708 (KAK2985842.1); *Actinidia chinensis var. chinensis*: Transcription factor like (PSS26124.1).


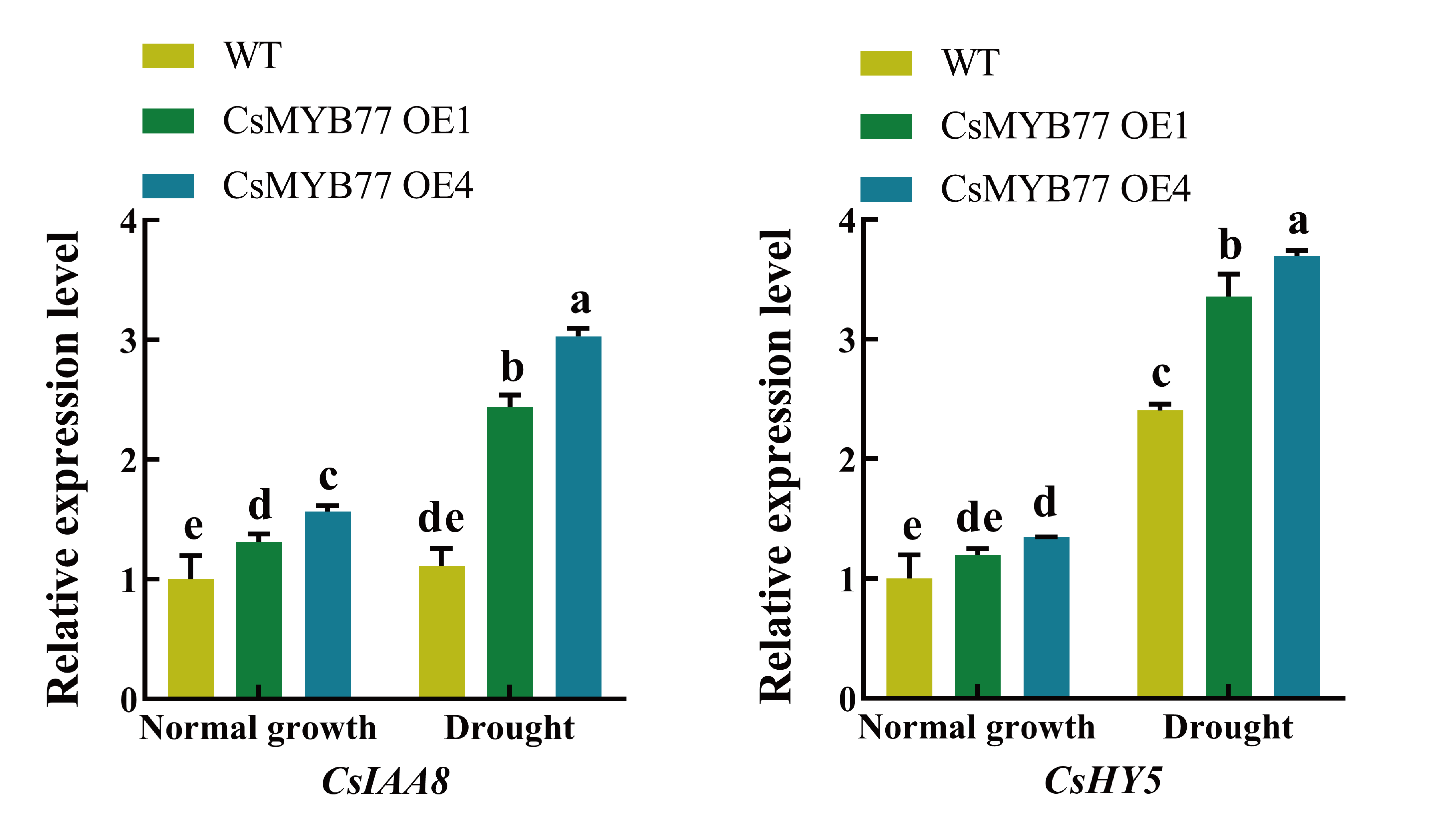


**Figure S7.** Relative expression levels of *CsIAA8* and *CsHY5* genes in overexpressed *Arabidopsis thaliana*. IAA8, indole-3-acetic acid; HY5, long hypocotyl 5. A one-way analysis of variance determined statistical significance. Statistical significance was determined using one-way analysis of variance (ANOVA). Different letters indicate statistically significant differences (*p* < 0.05). Error bars represent means ± SD.

**
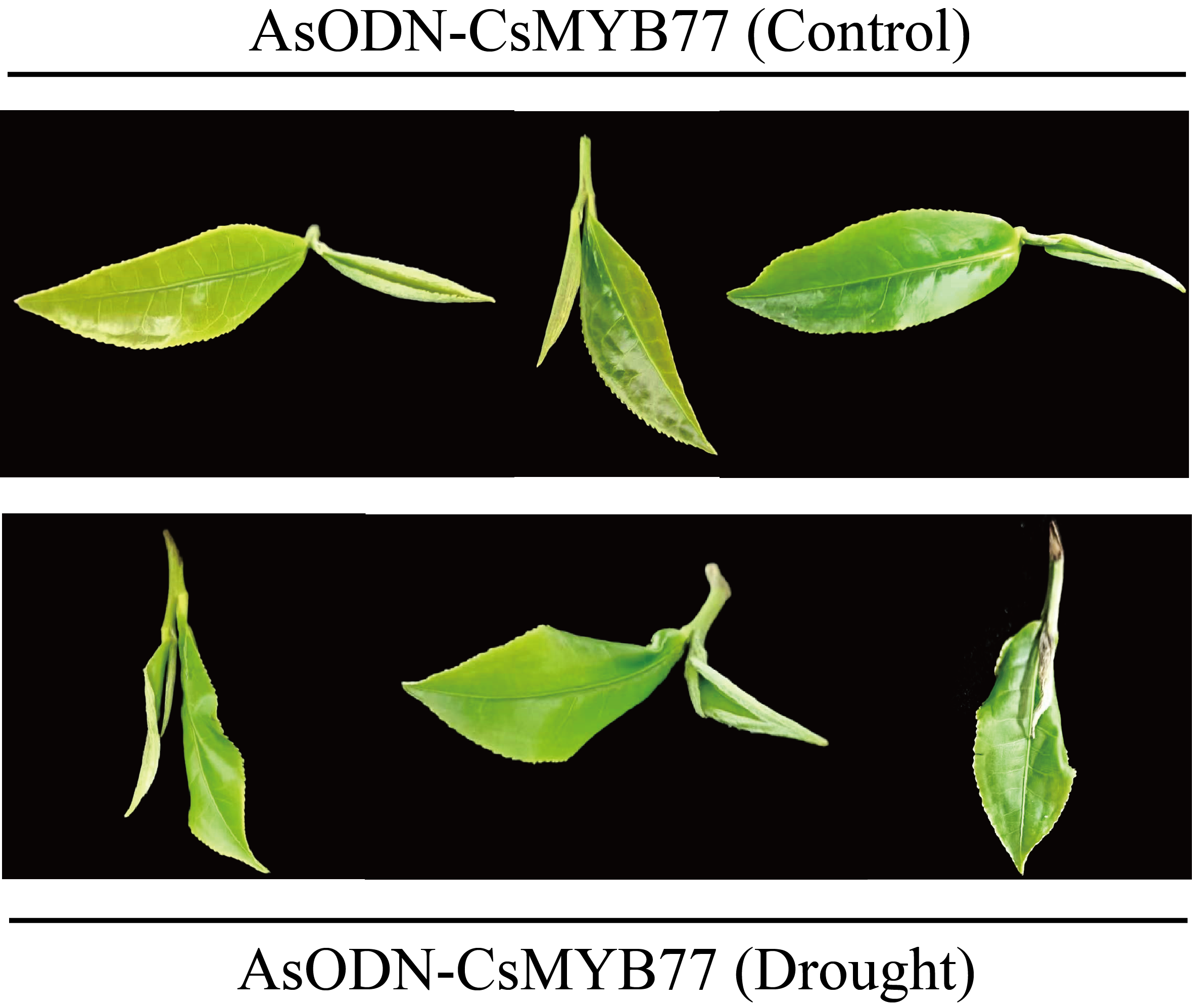
**

**Figure S8.** After silencing the *CsMYB77* gene in tea plant top buds, the phenotype changes of tea plant top buds after natural drought.

**
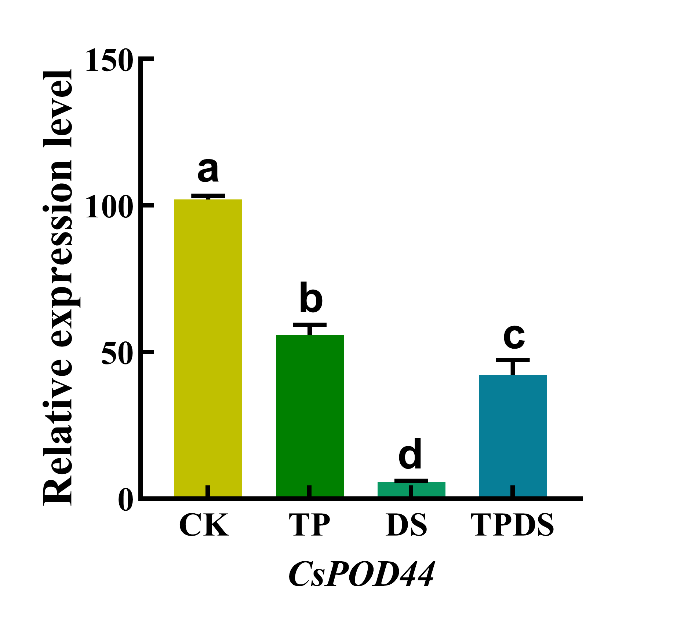
**

**Figure S9.** Relative expression changes of *CsPOD44* gene under four treatments. Statistical significance was determined using one-way analysis of variance (ANOVA). Different letters indicate statistically significant differences (*p* < 0.05). Error bars represent means ± SD.
