# Supplementary material for: Tea polyphenol mediated CsMYB77 regulation of CsPOD44 to promote tea plant (Camellia sinensis) root drought resistance
Source: Hortic Res. 2025 Feb 18;12(6):uhaf048. doi: 10.1093/hr/uhaf048 (PMC12010877; doi:10.1093/hr/uhaf048)
Supplement: Web_Material_uhaf048 [file web_material_uhaf048.zip › Supplemental tables.docx]

**Table S1**. Sequencing data quality assessment.

| Sample | Raw Reads | Clean Reads | Clean Base (G) | Error Rate (%) | Q20 (%) | Q30 (%) | GC Content (%) |
| --- | --- | --- | --- | --- | --- | --- | --- |
| CK-R_1 | 46338952 | 44859994 | 6.73 | 0.03 | 97.73 | 93.51 | 44.93 |
| CK-R_2 | 44352466 | 43028438 | 6.45 | 0.03 | 97.72 | 93.5 | 45.02 |
| CK-R_3 | 45215068 | 43330238 | 6.5 | 0.03 | 97.64 | 93.3 | 44.4 |
| DS-R_1 | 47559218 | 45721784 | 6.86 | 0.03 | 97.74 | 93.52 | 44.33 |
| DS-R_2 | 43669596 | 42620068 | 6.39 | 0.03 | 97.67 | 93.35 | 44.16 |
| DS-R_3 | 53639916 | 52207960 | 7.83 | 0.03 | 97.75 | 93.57 | 44.21 |
| TPDS-R_1 | 52034946 | 50145336 | 7.52 | 0.03 | 97.7 | 93.45 | 44.88 |
| TPDS-R_2 | 54904996 | 52219446 | 7.83 | 0.03 | 97.71 | 93.47 | 44.73 |
| TPDS-R_3 | 53548346 | 51504196 | 7.73 | 0.03 | 97.73 | 93.51 | 44.65 |
| TP-R_1 | 45170840 | 43675970 | 6.55 | 0.03 | 97.67 | 93.36 | 45 |
| TP-R_2 | 50161382 | 48096308 | 7.21 | 0.03 | 97.61 | 93.23 | 45.36 |
| TP-R_3 | 53888558 | 52212768 | 7.83 | 0.03 | 97.69 | 93.42 | 44.64 |

Note: CK-R, control group of roots; DS-R, drought stress on roots; TPDS-R, drought stress after exogenous tea polyphenols on roots; TP-R, exogenous tea polyphenols in the control group of roots; Raw Reads, the number of raw data reads; Clean Reads, the number of high-quality reads after filtering; Clean Base, the total number of bases in high-quality reads; Error Rate, sequencing error rate; Q20/Q30, the percentage of bases with a base accuracy of 99%/99.9% to the total bases; GC Content, the percentage of GCs to the total bases.

**Table S2**. primers used in this study

| Usage Primer | of name | Sequence (5’-3’) |
| --- | --- | --- |
| Subcellular localization | 35S:CsMYB77-GFP-F | caaattcgcgaccggtATGGAGAATCGATCATGGGTAATG |
|  | 35S:CsMYB77-GFP-R | tgctagtcataccggtCGGAGGAGGAGGAATTACCA |
| Overexpression Arabidopsis | P1300-CsMYB77-F | gtcccagactacgctggatccATGGAGAATCGATCATGGGTAATG |
|  | P1300-CsMYB77-R | gctcaccatggtaccggatccTTACGGAGGAGGAGGAATTACCA |
| Yeast one-hybrid assays | pGADT7-CsMYB77-F | gtgggcatcgatacgggatccATGGAGAATCGATCATGGGTAATG |
|  | pGADT7-CsMYB77-R | cagctcgagctcgatggatccTTACGGAGGAGGAGGAATTACCA |
|  | pHis-CsPOD44-F | gactcactatagggcgaattcGTTCATTTAAAAAAGAATCAATCATTTTAA |
|  | pHis-CsPOD44-R | gcgtgagctccccgggaattcAGTATTTTTTGAGAAGATGAGCCAATC |
| Dual-luciferase reporter assays | pGreen62-SK-CsMYB77-F | gccgctctagaactagtggatccATGGAGAATCGATCATGGGTAATG |
|  | pGreen62-SK-CsMYB77-R | ttggtaccgggccccccctcgagTTACGGAGGAGGAGGAATTACCA |
|  | pGreen-0800-LUC-CsPOD44-F | cactatagggcgaattgggtaccGTTCATTTAAAAAAGAATCAATCATTTTAA |
|  | pGreen-0800-LUC-CsPOD44-R | tatgtttttggcgtcttccatggAGTATTTTTTGAGAAGATGAGCCAATC |
| EMSA | pMAL-c6T-CsMYB77-F | cgcgatatcgtcgacggatccATGGAGAATCGATCATGGGTAATG |
|  | pMAL-c6T-CsMYB77-R | acctgcagggaattcggatccTTACGGAGGAGGAGGAATTACCA |
|  | CsPOD44-probe-F | GTTAAAATGGATAAGAATGTAACAGTTGACAAAAATAAAAAATTGAGCAT |
|  | CsPOD44-probe-R | ATGCTCAATTTTTTATTTTTGTCAACTGTTACATTCTTATCCATTTTAAC |
| As ODN | target sequence-1 | UAAUUCCUCCUCCUCCGUAA |
|  | antisense oligo-1 | TTACGGAGGAGGAGGAATTA |
|  | target sequence-2 | AUUUCGGAGAUGGUAAUUCC |
|  | antisense oligo-2 | GGAATTACCATCTCCGAAAT |
|  | target sequence-3 | CAAGUAAUAAGGCGAUUGGA |
|  | antisense oligo-3 | TCCAATCGCCTTATTACTTG |
|  | target sequence-4 | AAGAGGACGACCUCAUCCUU |
|  | antisense oligo-4 | AAGGATGAGGTCGTCCTCTT |
| DNA | CsMYB77-F | TCAGTATATCGCAGCCGATG |
|  | CsMYB77-R | CCACCACTCCACTGGCTACT |
| qRT-PCR | CsMYB77-F | GCGGACGAGTACTCTTGGAG |
|  | CsMYB77-R | GTGCTCCCCTTCGTACGTTA |
|  | CsPOD44-F | TGTCACAGCCAAACTTCGAG |
|  | CsPOD44-R | GATTGCGTAGTCTGCGTTGA |
|  | CsPAL-F | GGAGAGTGGAAATCCAACGA |
|  | CsPAL-R | ATCGATCATCTTCCCATTGC |
|  | CsCAD-F | TAACGGTTTTTCGGGTTGAG |
|  | CsCAD-R | TGCTCGAATCGTCTTCACAC |
|  | CsCOMT-F | GGAGTCACCCTCAGCATCAT |
|  | CsCOMT-R | GCTGTATGAGCTGCATCGAA |
|  | CsC4H-F | GCGTTGTTTGTGAAGCTGAA |
|  | CsC4H-R | GTCCTTGAAGAGCTGCAACC |
|  | CsCCR-F | TGTGTCGGAGTGTTCCATGT |
|  | CsCCR-R | AGCAGCTCCGGAAGATACAA |
|  | CsF5H-F | CCCATGGGATTCCTATTCCT |
|  | CsF5H-R | CCGTATTGTTTTGCCAGCTT |
|  | CsSOD-F | ATGGTTGCATGTCAACTGGA |
|  | CsSOD-R | GACAACAGCCCTCCCAATAA |
|  | CsAPX-F | AACAATGGCCTTGAGATTGC |
|  | CsAPX-R | CCCTTAGCAGCATCAGGAAG |
|  | CsMPK-F | CTTGCGGGTAAAGACCATGT |
|  | CsMPK-R | CATGTGGGAAAACCTTTGCT |
|  | AtPHYB-F | GGCTCCTCATGGTTGTCACT |
|  | AtPHYB-R | AGTGTGATGGCAAACAACCA |
|  | AtPIP-F | AGGTGGAGCCAACACCATAG |
|  | AtPIP-R | GGGGATGGTTGCTAAGTGAA |
|  | AtAPX-F | CTCTTGAGCGGAGAGAAGGA |
|  | AtAPX-R | GCAAACCCAAGCTCAGAAAG |
|  | AtSOD-F | TGGAGGAAAACCATCAGGAG |
|  | AtSOD-R | GGATTCACAGCATTGGGAGT |
|  | AtMPK-F | TCCATCAAATCATTCGGTCA |
|  | AtMPK-R | AGTTTGCGTTCAGGAGGAGA |
|  | AtPOD44-F | GCTTTGTCAGGGGTTGTGAT |
|  | AtPOD44-R | AATGCGACCGAGTCTCTTGT |
|  | AtCCR-F | TATGTGGATGTTCGCGATGT |
|  | AtCCR-R | GGTTCTTCTCGTCCTTGCAC |
|  | AtF5H-F | ATCAGATCCCACGACGTCTC |
|  | AtF5H-R | GCACAATCCGCCATACTTTT |
|  | AtCAD-F | TTTAGACCGGAAATCGATGG |
|  | AtCAD-R | CATATCCACCGCTCCTGAAT |
|  | At4CL-F | ATCGATCGGATTCTTCATCG |
|  | At4CL-R | AGTCCGTTGGTGAGATACGG |
|  | AtIAA8-F | GATTTCGGTTTGCTGAGTCC |
|  | AtIAA8-R | GATCCTTTCACACCCGAAAA |
| qRT-PCR | AtHY5-F | GTTTGGAGGAGAAGCTGTCG |
|  | AtHY5-R | TCTTGCTTGCTGAGCTGAAA |

Note: PAL, phenylalanine ammonia lyase; C4H, cinnamate-4 hydroxylase; 4CL, 4 coumarate CoA: Ligase; CAD, cinnamyl-alcohol dehydrogenase; F5H, ferulate-5-hydroxylase; CCR, cinnamoyl-CoA reductase; COMT, caffeic acid 3-O-methyltransferase; MPK, mitogen-activated protein kinase; PHYB, Phytochrome B; PIP, Plasma membrane intrinsic proteins; POD, peroxidase; SOD, superoxide dismutase; APX, ascorbate peroxidase; IAA, indole acetic acid; HY5, long hypocotyl 5.

**Table S3.** Changes in the content of catechin components in tea leaves under normal growth and drought stress, in the control group and after silencing *CsMYB77*

| mg/g(DW) | Normal growth | | Drought | |
| --- | --- | --- | --- | --- |
|  | Control | As ODN-*CsMYB77* | Control | As ODN-*CsMYB77* |
| EGC | 24.816±2.227b | 27.817±3.361b | 34.856±0.882a | 19.772±0.477b |
| DL-C | 4.728±0.201b | 4.809±1.086b | 7.121±0.422a | 6.044±0.459ab |
| EC | 8.584±0.432a | 7.702±1.676a | 9.963±0.570a | 9.055±1.137a |
| EGCG | 58.132±3.022b | 55.667±3.522b | 63.295±1.172a | 48.512±1.902c |
| GCG | 4.443±0.027a | 4.621±0.074a | 4.63±0.057a | 2.73±0.055b |
| ECG | 42.577±2.181b | 39.225±3.378b | 57.335±4.143a | 24.271±3.503c |
| Total catechin | 139.282±9.044b | 135.024±3.540b | 173.032±6.997a | 107.927±4.838c |

Note: C, catechin; EGC, (−)-epigallocatechin gallate; EC, epicatechin; EGCG, (−)-epigallocatechin gallate; ECG, (−)-epicatechin gallate; GCG, (−)-gallocatechin gallate. A one-way analysis of variance determined statistical significance. Different letters indicate significant differences (*p*<0.05).
